# NAT/NCS2-hound: a webserver for the detection and evolutionary classification of prokaryotic and eukaryotic nucleobase-cation symporters of the NAT/NCS2 family

**DOI:** 10.1093/gigascience/giy133

**Published:** 2018-11-10

**Authors:** A Chaliotis, P Vlastaridis, C Ntountoumi, M Botou, V Yalelis, P Lazou, E Tatsaki, D Mossialos, S Frillingos, G D Amoutzias

**Affiliations:** 1 Bioinformatics Laboratory, Department of Biochemistry and Biotechnology, University of Thessaly, Larisa, 41500, Greece; 2 Laboratory of Biological Chemistry, Department of Medicine, University of Ioannina, Ioannina, 45110, Greece; 3 Molecular Bacteriology Laboratory, Department of Biochemistry and Biotechnology, University of Thessaly, Larisa, 41500, Greece

**Keywords:** nucleobase transporters, nucleobase-ascorbate transporter (NAT) family, nucleobase-cation symporter-2 (NCS2) family, prokaryotes, evolution, MEME motifs

## Abstract

Nucleobase transporters are important for supplying the cell with purines and/or pyrimidines, for controlling the intracellular pool of nucleotides, and for obtaining exogenous nitrogen/carbon sources for metabolism. Nucleobase transporters are also evaluated as potential targets for antimicrobial therapies, since several pathogenic microorganisms rely on purine/pyrimidine salvage from their hosts. The majority of known nucleobase transporters belong to the evolutionarily conserved and ubiquitous nucleobase-ascorbate transporter/nucleobase-cation symporter-2 (NAT/NCS2) protein family. Based on a large-scale phylogenetic analysis that we performed on thousands of prokaryotic proteomes, we developed a webserver that can detect and distinguish this family of transporters from other homologous families that recognize different substrates. We can further categorize these transporters to certain evolutionary groups with distinct substrate preferences. The webserver scans whole proteomes and graphically displays which proteins are identified as NAT/NCS2, to which evolutionary groups and subgroups they belong to, and which conserved motifs they have. For key subgroups and motifs, the server displays annotated information from published crystal-structures and mutational studies pointing to key functional amino acids that may help experts assess the transport capability of the target sequences. The server is 100% accurate in detecting NAT/NCS2 family members. We also used the server to analyze 9,109 prokaryotic proteomes and identified Clostridia, Bacilli, β- and γ-Proteobacteria, Actinobacteria, and Fusobacteria as the taxa with the largest number of NAT/NCS2 transporters per proteome. An analysis of 120 representative eukaryotic proteomes also demonstrates the server's capability of correctly analyzing this major lineage, with plants emerging as the group with the highest number of NAT/NCS2 members per proteome.

## Introduction

The nucleobase-ascorbate transporter/nucleobase-cation symporter-2 (NAT/NCS2) protein family encompasses ion-gradient driven transporters of key metabolites or anti-metabolite analogs with diverse substrate preferences, ranging from purine or pyrimidine permeases in various organisms to Na^+^-dependent vitamin C transporters in human and other mammals [1–6]. Their additional function as providers of nitrogen/carbon may also affect energy production, replication, and protein synthesis through the salvage pathways for nucleotide synthesis [7–9]. In addition to their important direct role on the central metabolism of the cell, these and other nucleobase transporters have attracted interest as potential targets of purine/pyrimidine-based antimicrobials that could either be selectively routed into target cells to act as anti-metabolites or selectively inhibit an essential nucleobase transporter of the target cell [10–14].

This protein family is 1 of the 18 known families of the Amino acid-Polyamine-organoCation (APC) superfamily [15] and represents a subset of APC families that conform to a distinct structural/mechanistic pattern. The NAT/NCS2 transporters consist of 14 transmembrane segments (TMs) divided in two inverted repeats (7+7) and arranged spatially into a core domain (TMs 1–4 and 8–11) and a gate domain (TMs 5–7 and 12–14) [16]. The core domain contains all major determinants of the substrate-binding site, whereas the gate domain contributes to alternating access by allowing conformational rearrangements and providing major gating elements. The proteins probably function as homodimers and may use an elevator-like mechanism to achieve alternating access [17, 18]. Similar structural features are described for transporters of two other APC families, the sulfate permeases (SulP) [19] and the anion exchangers (AE), which include the well-studied chloride/bicarbonate exchanger (band 3) of human erythrocytes [20].

The NAT/NCS2 is split phylogenetically in two subfamilies. The first one, COG2233 or NAT, contains bacterial and fungal permeases for purines (xanthine, uric acid), bacterial permeases for pyrimidines (uracil, thymine), plantal and mammalian broad-specificity uracil/purine permeases (not present in human), and the mammalian L-ascorbate transporters SVCT1 and SVCT2. Insight on the transport mechanism of this subfamily has been provided by high-resolution crystal structures for two members, the uracil permease UraA of *Escherichia coli* [16, 18] and the xanthine/uric acid permease UapA of *Aspergillus nidulans* [17], coupled with extensive mutagenesis studies on UapA [21], the xanthine permease XanQ of *E. coli* [1, 22], and few other homologs [23, 24]. The other subfamily, COG2252 or AzgA-like [25], contains bacterial, fungal, and plantal permeases for salvaegable purines (adenine, guanine, hypoxanthine), which are less well studied with respect to structure-function relationships [7, 26].

Despite their importance, membrane transporters, in general, and the NAT/NCS2 family, in particular, are not so extensively studied to date as other categories of proteins are due to the inherent difficulties in experimentation and in accurate prediction of their function [27, 28]. Based on a large-scale evolutionary analysis that we performed in this study, we have identified in prokaryotes the major evolutionary groups and subgroups, with distinct substrate specificities; identified key motifs for each phylogenetic group and subgroup that are related to substrate specificity; developed a webserver that utilizes all the above information to detect and classify at proteome-scale NAT/NCS2 transporters; and analyzed with this webserver 9,109 prokaryotic and 120 Eukaryotic proteomes so as to investigate which evolutionary lineages are rich in these transporters. We expect that this type of analyses and the accompanying computational tool, which are lacking in general for other families of transporters, will facilitate the experimental study of new homologs, provide a practical tool for assignment of homologs into functionally relevant associated subgroups, and also improve their annotation in the databases.

## Materials and Methods

### Development of hidden Markov models and Meme motifs for the family, subfamilies, and evolutionary clusters

All the annotated sequences of the 2A APC superfamilies (organized into 18 families) were obtained from the transporter classification database (TCDB) [15]. For each of the 18 families, we generated protein alignments with Muscle and SeaView [29, 30] that were manually edited and then used to build a hidden Markov model (HMM) for each one with HMMER [31].

Next, 4,442 bacterialand 213 archaeal proteomes were downloaded from UNIPROT (January 2017) [32]. Their protein sequences were scanned with the above 18 HMMs and, thus, 8,291 proteins of the NAT/NCS2 family were identified and retained for further analysis. Then, close homologs were removed with the Blastclust software, using as cutoff 70% protein identity over 70% of sequence length. Thus 1,355 NAT/NCS2 sequences were retained after this step.

Subsequently, these sequences were fed to the MEME software [33] so as to identify 14 motifs of length 14–21 or 18–25 amino acids each. Manual inspection of sequences with a very low number of motifs resulted in rejection of 14 sequences. Thus, 1,341 sequences were retained. These 1,341 sequences were scanned again with the 14 MEME motifs by MAST [33]. Custom Perl scripts were developed to obtain the motif presence/absence for each sequence as a vector of 0 and 1 values, based on detection with MAST (see Supplementary Materials Custom_scripts). The above vectors were clustered in MATLAB with the Clustergram function (default parameters—commands found in Supplementary Materials Custom_scipts). This first round of clustering revealed two major evolutionary subfamilies, designated SF1 and SF2 (see Supplementary Fig. S1). The sequences of each subfamily were fed to another round of MEME-motif detection with the same parameters as in the first instance. Again, 14 MEME motifs were made for each subfamily. These were used with the MAST software to identify MEME-Motif content for each subfamily; again, vectors of motif presence/absence were generated for each subfamily (and their clusters were manually inspected; see Supplementary Figs. S2 and S3). All Meme/Mast results and analyzed sequences are found in the Supplementary Materials “MEME_MAST_motifs.”

Next, the protein sequences of each subfamily were aligned and manually edited separately with Muscle and Seaview [29, 30]. Furthermore, in each subfamily, sequences with experimental evidence of substrate specificity were added (eukaryotic ones as well). Phylogenetic trees were generated with the BioNJ method using the Poisson model and 1,000 bootstraps. The two generated phylogenetic trees (for each of the two distinct subfamilies; see Supplementary Figs. S4 and S5) were annotated and visualized in Archaeopteryx and Treedyn [34, 35]. Subfamily 1 was organized into six major and four very small clusters. Subfamily 2, which was more homogeneous than subfamily 1, was organized into many small clusters. HMMs were thus constructed for the NAT/NCS2 family, its two subfamilies, and each of the six major clusters in subfamily 1. For several of the small clusters in subfamily 2 that contained sequences with known substrates, we also generated extra HMMs. In addition, we generated 14 MEME motifs for each subfamily and each of the six clusters in subfamily 1. A workflow of how the various HMMs and MEME motifs were generated is found in Supplementary Fig. S12. All edited sequence alignments, HMMs, and phylogenetic trees (in newick format) are organized in Supplementary Materials “Sequence_alignments_HMMs_phylogenetic_trees.”

### Development and evaluation of the server

All of the above HMMs and MEME motifs were incorporated into a webserver, named NAT/NCS2-hound, that may scan protein sequences in FASTA format, identify members of this family, and further classify them in the various subfamilies and clusters. The webserver is based on the Jhipster Application Framework [36] that utilizes Angular Javascript Framework for the front end and the Java language and Spring Framework for the back end. The server is freely available at [37].

The server and instructions for local installation are found in the Supplementary Material “Server_for_local_installation.” Also, the server is registered at SciCrunch.org with RRID:SCR_016473.

Functional information for the various amino acids was obtained from several mutational studies [1, 21, 24] and from the structural studies on UraA [16, 18] and UapA [17].

We performed an evaluation analysis in order to assess the effectiveness of the NAT/NCS2-hound server. TCDB-annotated transporters of the 18 families of the APC superfamily were used as bait to obtain best blast hits against bacterial reference proteomes downloaded from Uniprot. The best blast hit of a bait sequence was designated as a member of the family that its annotated (from TCDB) bait sequence belonged to. These retrieved best blast hit sequences constituted the evaluation set. Any of these sequences that had been used to train the HMMs were removed from the evaluation set. Thus, we retrieved/retained 7,799 APC sequences, of which 975 belonged to the NAT/NCS2 family. These were scanned by our server for detection and evolutionary classification. The server demonstrated 100% accuracy (100% sensitivity and 100% specificity) in detecting NAT/NCS2 family members and can further categorize them to the various evolutionary subgroups and display conserved motifs and relevant functional information/annotation.

In order to assess the distribution of NAT/NCS2 family in major taxonomic lineages, 9,109 prokaryotic proteomes (downloaded from the National Center for Biotechnology Information [NCBI] in March 2018) and 120 eukaryotic reference proteomes (downloaded from Uniprot in March 2018) were scanned by our server. The presence of a minimum number of seven MEME motifs was required as a cutoff to filter out any sequence fragments. All results and sequence IDs are found in Supplementary Tables S1–S6.

## Results and Discussion

### The NAT/NCS2 family is organized into two major subfamilies

An analysis of 1,341 proteins, based on the presence/absence of conserved MEME motifs within the NAT/NCS2 family, clearly revealed the presence of two distinct and major subfamilies (see Supplementary Fig. S1). Previous phylogenetic analyses also revealed the presence of these two major subfamilies [7], in accordance with the presence of two COGs, designated as COG2233 (xanthine/uracil permease) and COG2252 (AzgA-like). Subfamily 1 (COG2233) consisted of 748 sequences and subfamily 2 (COG2252, AzgA-like) consisted of 593 sequences. The members of subfamily 1 display a greater degree of sequence divergence among them, whereas members of subfamily 2 constitute a more homogeneous set of sequences (Supplementary Figs. S2 and S3).

### Distinct phylogenetic clusters within subfamily 1

**Figure 1:**
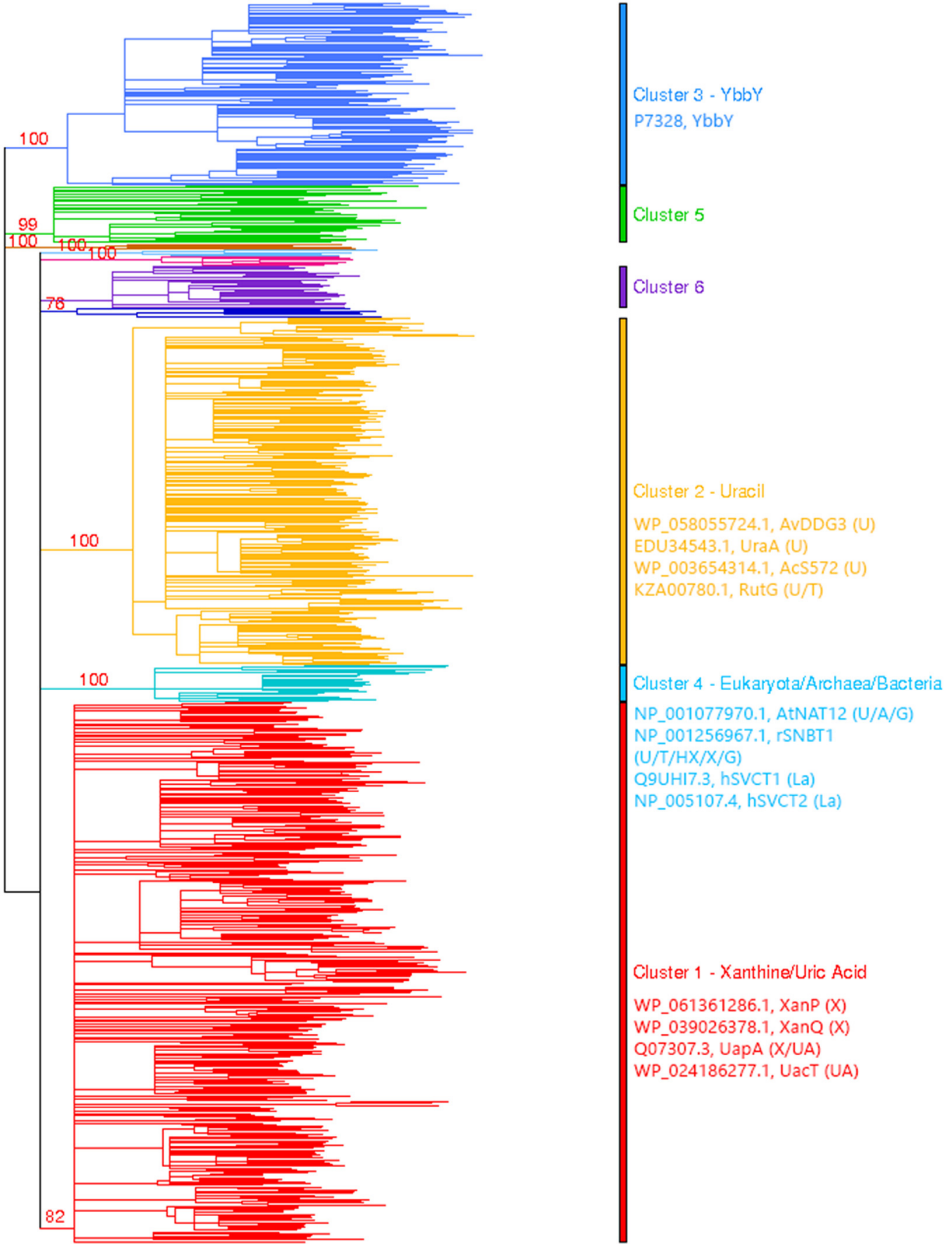
Phylogenetic tree of subfamily 1 of the NAT/NCS2 family. The various phylogenetic clusters are depicted with different colors. Sequence redundancy was removed at a cutoff of 70% protein identity over 70% of sequence length. Well-characterized, known homologs are indicated with their major substrates in parenthesis (U, uracil; T, thymine; A, adenine; G, guanine; HX, hypoxanthine; La, L-ascorbic acid; X, xanthine, UA, uric acid).

Further phylogenetic analysis of the more diverse members of subfamily 1 reveals the clear presence of six major clusters (clusters 1–6) and four minor clusters (see Fig. 1). The incorporation of functionally annotated sequences from all kingdoms of life further helped us understand the substrate specificity profile of each cluster whenever relevant information for representative homologs was available. The largest and most diverse cluster (cluster 1) contains sequences that have been annotated to transport xanthine or uric acid or both. The second largest cluster (cluster 2) contains sequences that are known to transport uracil or uracil and thymine. The third largest cluster (cluster 3) contains the YbbY gene from *E. coli*, a homolog that is not functionally annotated in the databases, but recent evidence suggests that it transports adenine, guanine, and hypoxanthine (Botou and Frillingos, unpublished data). The fourth cluster (cluster 4) contains functionally characterized sequences from eukaryotes but also encompasses functionally unknown homologs from archaea as well as a few bacteria. All the other clusters do not contain any sequences of known function.

Subfamily 2 is more homogeneous and is organized in many small clusters, with small differences among them. For several of those small clusters that contain sequences with known substrates, we generated additional HMMs. A more detailed inspection of the various phylogenetic trees for each subfamily and each of the major six clusters within subfamily 1 are available in the Supplementary Figs. S4–S11.

### A web server for the detection and evolutionary classification of NAT/NCS2 family members

Of aAll the above evolutionary analyses, the HMMs and MEME motifs generated for the various subfamilies and evolutionary clusters have been used to develop a webserver for the detection and evolutionary classification of NAT/NCS2 family members, named NAT/NCS2-hound. The webserver is freely available at [37]. This webserver detects and distinguishes this family of transporters from the other 17 homologous families of the APC superfamily. Furthermore, it can categorize these transporters to certain subfamilies and clusters associated with distinct substrate specificities based on the large-scale phylogenetic analysis that we performed on prokaryotic proteomes. For each one separately, the identified set of characteristic signature motifs is detected. Furthermore, for several key subgroups, we have integrated information from published crystal structures and mutational studies to help experts identify key functional amino acids and help them assess the transport capability of the scanned sequences. Nevertheless, this server does not function as a prediction tool of substrate specificity. The NAT/NCS2-hound server implements for this important family the same principles and computational protocol that were developed/implemented recently for another prokaryotic superfamily, the tRNA-synthetases [38].

**Figure 2:**
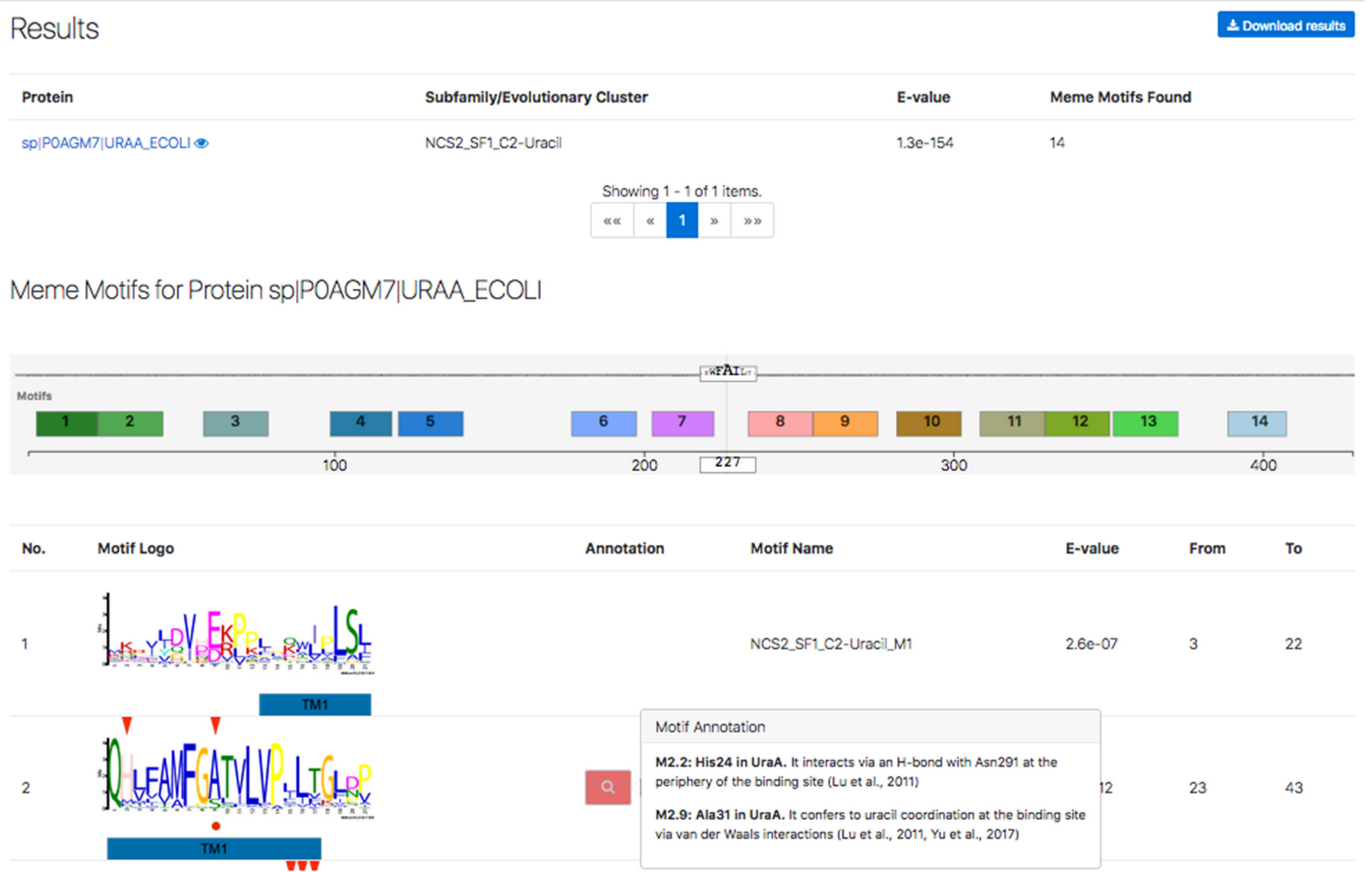
Display of results from the NAT/NCS2-hound server, including the best HMM that detects the protein sequence, the various MEME conserved motifs, and any available functional information/annotation for specific sites in certain motifs.

The input for this server is a protein sequence or a proteome file in FASTA format. The webserver displays graphically (see Fig. 2) which proteins have been identified as NAT/NCS2, to which subfamily and cluster they belong to, and which conserved motifs have been identified on the target proteins. For several key subgroups and motifs, the server further displays annotated (by our experts) information from published crystal structures and mutational studies pointing to key functional amino acids of well-studied representative homologs.

The server has been evaluated against a dataset of 7,800 homologous transporters of the APC superfamily, of which 975 belong to the NAT/NCS2 family and displayed 100% accuracy (100% sensitivity and 100% specificity).

### Distribution of the NAT/NCS2 family and subfamilies in prokaryotes and eukaryotes

In order to assess the distribution of NAT/NCS2 members in major taxonomic lineages, we scanned 9,109 prokaryotic proteomes (from NCBI) and 120 representative eukaryotic proteomes (from Uniprot). As a filter, we only included in our analysis 29,096 prokaryotic and 361 eukaryotic NAT/NCS2 proteins that had at least seven Meme motifs each in order to exclude small sequence fragments.

**Figure 3:**
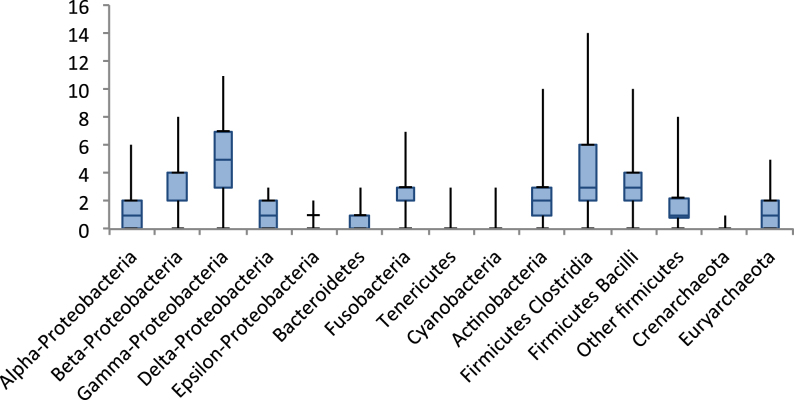
Box plot of the distribution of NAT/NCS2 proteins per proteome in major prokaryotic taxonomic lineages. The analysis was based on 9,109 bacterial and archaeal proteomes. β and γ-Proteobacteria, Clostridia, Bacilli, Fusobacteria, and Actinobacteria had the highest numbers of transporters per proteome (on average).

We found that 80% (7,318/9109) of the prokaryotic proteomes had at least one NAT/NCS2 protein based on our criteria, with four NAT/NCS2 proteins, on average. The lineages with the most proteins (on average) are Clostridia, Bacilli, β- and γ-Proteobacteria, and Actinobacteria (see Fig. 3). The proteomes with the most transporters were *Clostridium bolteae* (with 14 members), several different γ-Proteobacteria (*Morganella morganii, Enterobacter lignolyticus, Citrobacter amalonaticus*), and *Bacillus megaterium* with 11 members each, followed by many other γ-Proteobacteria (such as *E. coli*) with 10 members each (see Supplementary Tables S1 and S2). Subfamily 1 was more abundant than subfamily 2, comprising of 58% of all NAT/NCS2 proteins (paired *t*test *P* value = 0). Also, cluster 1 (xanthine/uric acid) and cluster 2 (uracil) from subfamily 1 comprised of 29% and 23% of the total number of NAT/NCS2 proteins detected, whereas cluster 3 (YbbY) was the third largest one, comprising of 5% of the total number of NAT/NCS2 proteins.

A total of 637 of the prokaryotic species included in this analysis were represented by at least two strains each, of which 97 species had 10–50 strains, 10 species with 50–100 strains, and 9 species had more than 100 strains (see Supplementary Table S3, strain volatility.csv). For 73% (463/637) of these studied species, there was no change in the number of NAT/NCS2 proteins among the various strains of a certain species. Striking examples are four species with more than 100 strains each (*Staphylococcus aureus, Listeria monocytogenes, Mycobacterium tuberculosis, Campylobacter jejuni*) that displayed no difference in the numbers of NAT/NCS2 proteins among different strains. On the other end, the most notable exceptions were *Streptomyces hygroscopicus* (4 strains with 3–9 homologs per strain), *Clostridium botulinum* (34 strains with 5–10 homologs), *Pseudomonas fluorescens* (16 strains with 4–8 homologs), and *Pseudomonas stutzeri* (10 strains, with 3–7 homologs).

The number of NAT/NCS2 proteins per strain ranged from 14 to 0 (see Supplementary Tables S2 and S3). Most strains that contain no NAT/NCS2 homologs belong to parasitic and/or endosymbiotic bacteria (*Chlamydia*, *Rickettsia*, *Ehrlichia*, *Wolbachia*, *Coxiella*, *Leptospira*, *Capnocytophaga*, *Cellulophaga*, *Bartonella*, *Mycoplasma*, *Mycobacterium* (17/40 species), *Flavobacterium* (4/8 species), *Xanthomonas oryzae*, *Xylella fastidiosa*, *Mesorhizobium* (5/6 species), *Rhizobium* (4/5 species), *Liberibacter*, *Buchnera aphidicola*), or autotrophic bacteria or archaea with specialized metabolic adaptations (*Sulfolobus*, *Metallosphaera*, *Aquifex*, *Sulfurimonas*, *Halothiobacillus*, *Dehalococcoides*, *Oligotropha*, *Methanosarcina* (7/8 species), *Methanobacterium* (4/5 species), *Prochlorococcus*, *Rhodobacter*, *Rhodopseudomonas*). It is of interest that some known pathogenic bacteria that rely on purine salvage for nucleotide biosynthesis and infectivity [13, 39–41] retain few NAT/NCS2 homologs, all of which belong to subfamily 2 (classified as adenine/guanine/hypoxanthine transporters) (*Helicobacter pylori*, 85/85 strains, 1 homolog; *Borrelia burgdorferi*, 8/9 strains, 2 homologs). On the other end, 2,644 strains (29% of the total) that are distributed in 546 species (17% of the total) had 5 or more NAT/NCS2 homologs, with an average of 6 homologs per species (3.5–3.7 in subfamily 1 and 2.3–2.4 in subfamily 2). All of these strains are bacterial and most belong to heterotrophic, metabolically versatile species; their NAT/NCS2 homologs are distributed in several phylogenetic clusters, indicating a range of different nucleobase preferences. As an example, *E. coli* K-12, which has been studied thoroughly with respect to the relevant functional profiles, contains 10 NAT/NCS2 proteins, including the xanthine-specific transporters XanP and XanQ [42] and the uric acid transporter UacT [23] in cluster 1 (see Fig. 1), the uracil-specific UraA and the broader-specificity uracil/thymine transporter RutG [24] in cluster 4, the purine transporter YbbY in cluster 3, and two pairs of closely related transporters specific for adenine (AdeP, AdeQ) or guanine and hypoxanthine (GhxP, GhxQ) [7] that belong to subfamily 2. This versatility in substrate profiles is associated with different metabolic pathways and linkage of the transporter genes with different catabolic or biosynthetic operons [1, 24].

Furthermore, we did a survey on 61 proteomes from strains found in the human microbiome [43] and found that NAT/NCS2 homologs are enriched (three per genome, on average) in bacteria of the gastrointestinal tract (see Supplementary Table S4). We also analyzed 120 representative proteomes from animals, fungi, plants, and various unicellular Eukaryotes. We found that 83% of the detected proteins belonged to subfamily 1 (the rest to subfamily 2), with the vast majority (66% of the total) in cluster 4, followed by cluster 1 (14% of the total) and cluster 2 (3% of the total) (see Supplementary Tables S5 and S6 for detailed results and analyzed sequences). As an additional validation step, our server detected and properly classified well-known and previously annotated NAT/NCS2 sequences in certain selected species, such as the human homologs SVCT1 and SVCT2 [5], the rat uracil/purine transporter rSNBT1 [4], the uracil/purine transporters AtNAT3 and AtNAT12 and adenine/guanine transporters AtAzg1 and AtAzg2 of *Arabidopsis thaliana* [3], and the xanthine/uric acid transporters UapA and UapC and adenine/guanine/hypoxanthine transporter AzgA of *Aspergillus nidulans* [2]. Thus, although the development of the server was based on prokaryotic sequences, the server can successfully analyze eukaryotic sequences as well. This is attributed to the fact that all eukaryotic sequences are fully contained within evolutionary groups that are already present in prokaryotes. Plants had the highest number of NAT/NCS2 members (10 per genome on average), followed by animals (3 on average), and then fungi (2 on average), whereas most (73%) of the unicellular eukaryotes had no NAT/NCS2 proteins. Intriguingly, Metazoa had only sequences that belonged to cluster 4 (of subfamily 1). Plants also displayed a great expansion in members of cluster 4 but they also had small numbers of sequences from cluster 1 and subfamily 2 (AzgA-like). Fungi had a rather balanced number of sequences from cluster 1 and subfamily 2. Notably, tobacco (*Nicotiana tabacum)* had the most (30) NAT/NCS2 proteins, the fungus *Basidiobolus meristosporus* had 16 members, whereas two lophotrochozoa (*Lingula unguis*and*Crassostrea gigas*) had 12 members each. Although it is conceivable that the percentages observed in this analysis may change depending on the species sampling, the general trends observed for each major taxonomic lineage are expected to hold.

## Conclusions

Based on a large-scale phylogenetic analysis of prokaryotic NAT/NCS2 proteins, a webserver has been developed that can scan whole proteomes and identify members of this family. The server classifies these members in various subfamilies and evolutionary clusters with certain substrate profiles and identifies conserved motifs that are related to function. An analysis of 9,109 prokaryotic proteomes with our server revealed that the evolutionary lineages containing the largest numbers of NAT/NCS2 members are β- and γ-Proteobacteria, Bacilli, Clostridia, Actinobacteria, and Fusobacteria. An analysis of 120 eukaryotic proteomes also revealed that this server is fully capable of successfully analyzing this taxonomic lineage as well.

## Availability of source code and requirements

Project name: NAT-NCS2

Project home page: https://github.com/pvlastaridis/nat-ncs2

Operating system(s): Platform independent

Programming language: Java, Angular, Spring

Other requirements: Java JDK8, NodeJS 8.10, Yarn(yarnpkg.com) 1.10, Hmmer(http://hmmer.org/) 3.1

License: MIT

RRID:SCR_016473

## Availability of supporting data

All supplementary data can be downloaded from the NAT/NCS2-hound server at: http://bioinf.bio.uth.gr/nat-ncs2/. Additional supporting data and snapshots of the code are openly available in the *GigaScience* repository, GigaDB [44].

## Abbreviations

AE: anion exchanger; APC; HMM: hidden Markov model; NAT/NCS2: nucleobase-ascorbate transporter/nucleobase-cation symporter-2; NCBI: National Center for Biotechnology Information; TM: transmembrane segment; SulP: sulfate permeases; TCDB: transporter classification database.

## Competing interests

The authors declare that they have no competing interests.

## Funding

G.D.A. acknowledges support from the ARISTEIA II Action of the Operational Programme Education and Lifelong Learning that is co-funded by the European Social Fund and National Resources (grant 4288). G.D.A. also acknowledges support by research grants from the Postgraduate Program Applications of Molecular Biology-Genetics, Diagnostic Biomarkers [code 3817] of the University of Thessaly, School of Health Sciences, Department of Biochemistry and Biotechnology.

## Author contributions

A.C. and C.N. performed the phylogenetic analysis; P.V. developed the server; M.B., V.Y., P.L., and E.T. gathered annotation and functional information; D.M., S.F., and G.D.A. conceived the study, supervised the students, and prepared the manuscript.

## Supplementary Material

GIGA-D-18-00192_Original_submission.pdf

GIGA-D-18-00192_Revision_1.pdf

GIGA-D-18-00192_Revision_2.pdf

Response_to_Reviewer_Comments_Original_Submission.pdf

Response_to_Reviewer_Comments_revision_1.pdf

Reviewer_1_Report_(Original_Submission) -- Hong Qu 7/1/2018 Reviewed

Reviewer_1_Report_(Revsion1) -- Hong Qu 9/24/2018 Reviewed

Reviewer_2_Report_(Original_Submission) -- Yurong Wen 7/19/2018 Reviewed

Reviewer_2_Report_(Revsion1) -- Yurong Wen 9/29/2018 Reviewed
