# Supplementary material for: NAT/NCS2-hound: a webserver for the detection and evolutionary classification of prokaryotic and eukaryotic nucleobase-cation symporters of the NAT/NCS2 family
Source: Gigascience. 2018 Nov 10;7(12):giy133. doi: 10.1093/gigascience/giy133 (PMC6308229; doi:10.1093/gigascience/giy133)
Supplement: GIGA-D-18-00192_Revision_2.pdf [file giy133_giga-d-18-00192_revision_2.pdf]

# GigaScience

## NAT/NCS2-hound: A webserver for the detection and evolutionary classification of prokaryotic and eukaryotic nucleobase-cation symporters of the NAT/NCS2 family.

--Manuscript Draft--

| | |
|---|---|
| **Manuscript Number:** | GIGA-D-18-00192R2 |
| **Full Title:** | NAT/NCS2-hound: A webserver for the detection and evolutionary classification of prokaryotic and eukaryotic nucleobase-cation symporters of the NAT/NCS2 family. |
| **Article Type:** | Technical Note |
| **Funding Information:** | General Secretariat of Research and Technology of Greece (Aristeia II: 4288) — Dr Grigorios D Amoutzias<br>University of Thessaly, Greece (3817) — Dr Grigorios D Amoutzias |
| **Abstract:** | Nucleobase transporters are important for supplying the cell with purines and/or pyrimidines, for controlling the intracellular pool of nucleotides and for obtaining exogenous nitrogen/carbon sources for the metabolism. Nucleobase transporters are also evaluated as potential targets for antimicrobial therapies, since several pathogenic microorganisms rely on purine/pyrimidine salvage from their hosts. The majority of known nucleobase transporters belong to the evolutionarily conserved and ubiquitous NAT/NCS2 protein family. Based on a large-scale phylogenetic analysis that we performed on thousands of prokaryotic proteomes, we have developed a webserver that can detect and distinguish this family of transporters from other homologous families that recognize different substrates. We can further categorize these transporters to certain evolutionary groups with distinct substrate preferences. The webserver scans whole proteomes and graphically displays which proteins are identified as NAT/NCS2, to which evolutionary groups and subgroups they belong to and which conserved motifs they have. For key subgroups and motifs, the server displays annotated information from published crystal-structures and mutational studies pointing to key functional amino acids that may help experts assess the transport capability of the target sequences. The server is 100% accurate in detecting NAT/NCS2 family members. We also used the server to analyze 9109 prokaryotic proteomes and identified Clostridia, Bacilli, β- and γ-Proteobacteria, Actinobacteria and Fusobacteria as the taxa with the largest number of NAT/NCS2 transporters per proteome. An analysis of 120 representative eukaryotic proteomes also demonstrates the server's capability of correctly analyzing this major lineage, with plants emerging as the group with the highest number of NAT/NCS2 members per proteome. |
| **Corresponding Author:** | Grigorios D Amoutzias, Ph.D<br>University of Thessaly<br>Larisa, Thessaly GREECE |
| **Corresponding Author Secondary Information:** | |
| **Corresponding Author's Institution:** | University of Thessaly |
| **Corresponding Author's Secondary Institution:** | |
| **First Author:** | Anargyros Chaliotis |
| **First Author Secondary Information:** | |
| **Order of Authors:** | Anargyros Chaliotis |
| | Panayotis Vlastaridis |
| | Chrysoula Ntountoumi |
| | Maria Botou |
| | Vasilis Yalelis |
| | Panayota Lazou |

Powered by Editorial Manager® and ProduXion Manager® from Aries Systems Corporation

| | |
|---|---|
| | Ekaterini Tatsaki |
| | Dimitris Mossialos, Ph.D |
| | Stathis Frillingos |
| | Grigorios D Amoutzias, Ph.D |
| **Order of Authors Secondary Information:** | |
| **Response to Reviewers:** | GIGA-D-18-00192R1<br>NAT/NCS2-hound: A webserver for the detection and evolutionary classification of prokaryotic and eukaryotic nucleobase-cation symporters of the NAT/NCS2 family.<br>Anargyros Chaliotis; Panayotis Vlastaridis; Chrysoula Ntountoumi; Maria Botou; Vasilis Yalelis; Panayota Lazou; Ekaterini Tatsaki; Dimitris Mossialos, Ph.D; Stathis Frillingos; Grigorios D Amoutzias, Ph.D<br>GigaScience<br><br><br>Dear Dr. Nogoy,<br><br>Thank you very much for your very positive decision and the constructive criticism we have received. We now provide a revised version of our manuscript and the supplementary data as requested by Chris Armit, for inclusion in GigaDB.<br><br>We hope that the revised version of our manuscript now addresses these essential minor formatting revisions that you have suggested/requested. We provide detailed answers to the points raised, further bellow.<br><br>We are looking forward hearing from you at your earliest convenience.<br><br>With kind regards,<br><br>Grigoris Amoutzias and Stathis Frillingos, Corresponding Authors<br><br>Grigoris Amoutzias<br>Assistant Professor of Bioinformatics in Genomics<br>Department of Biochemistry and Biotechnology<br>University of Thessaly<br>Larisa, 41500, Biopolis<br>Greece<br><br><br>Dear Dr Amoutzias,<br><br>Your manuscript "NAT/NCS2-hound: A webserver for the detection and evolutionary classification of prokaryotic and eukaryotic nucleobase-cation symporters of the NAT/NCS2 family." (GIGA-D-18-00192R1) has been assessed by our reviewers. Based on these reports, and my own assessment as Editor, I am pleased to inform you that it is potentially acceptable for publication in GigaScience, once you have carried out some essential minor formatting revisions.<br><br>1) include a diagram showing the steps in the creation of the HMMs and MEME motifs. This could also fit in protocols.io - and add the protocols.io citation to the paper.<br><br>Answer: We now provide a supplementary figure S12 that includes the workflow diagram that you have requested. We also mention this within the manuscript:<br>“A workflow of how the various HMMs and MEME motifs were generated is found in supplementary figure S12_workflow_diagram.”<br>Concerning the protocols.io, subject, we believe/hope that the details within the Materials and Methods and the newly added workflow diagram are sufficient and protocols.io would be redundant.<br><br>2) move all URLs in the main text to the references and only cite the reference number in the main text. |

| | |
|---|---|
| | Answer: We have now moved the URLs to the references and only cite the reference number in the main text.<br>“36. JHipster - Generate your Spring Boot + Angular/React applications! [Internet]. [cited 2018 Oct 9]. Available from: https://www.jhipster.tech/<br>37. Prediction and Evolutionary Classification Server of prokaryotic and eukaryotic NAT/NCS2 transporters [Internet]. [cited 2018 Oct 9]. Available from: http://bioinf.bio.uth.gr/nat-ncs2/<br>43. NIH Human Microbiome Project - Project Catalog [Internet]. [cited 2018 Oct 9]. Available from: https://www.hmpdacc.org/catalog/”<br><br>3) include a "Availability of supporting source code and requirements" section (before the data availability section)<br><br>List the following:<br><br>Project name: e.g. My bioinformatics project<br>Project home page: e.g. https://github.com/ISA-tools<br>Operating system(s): e.g. Platform independent<br>Programming language: e.g. Java<br>Other requirements: e.g. Java 1.3.1 or higher, Tomcat 4.0 or higher<br>License: e.g. GNU GPL, FreeBSD etc.<br>RRID: if applicable, e.g. RRID: SCR_014986<br><br>This needs to be under an Open Source Initiative approved license where practicable compiled running software is made available. If the code is not hosted in a repository the GigaScience GitHub repository is also available for this purpose.<br>Their reports, together with any other comments, are below. Please also take a moment to check our website at https://giga.editorialmanager.com/ for any additional comments that were saved as attachments.<br><br>In addition, please register any new software application in the SciCrunch.org database to receive a RRID (Research Resource Identification Initiative ID) number, and include this in your manuscript. This will facilitate tracking, reproducibility and re-use of your tool.<br><br>Answer: We now provide this section, as requested.<br>Please note that we have also made some extra changes within the manuscript.<br>We have now acknowledged two sources of funding, that will cover our article processing costs.<br>In addition, Chris Armit from GigaDB has requested some modifications concerning supplementary tables that need to be provided as csv files and not excel files. We now mention these supplementary data within the manuscript accordingly.<br>“All results and sequence IDs are found in supplementary tables 1-6”<br>“…followed by many other γ-Proteobacteria (such as E.coli) with 10 members each (see supplementary tables 1 & 2).”<br>“10 species with 50-100 strains and 9 species had over 100 strains (see supplementary_table_3_strain_volatility.csv).”<br>“The number of NAT/NCS2 proteins per strain ranged from 14 to 0 (see supplementary tables 2 & 3).”<br>“… in bacteria of the gastrointestinal tract (see supplementary table 4).”<br>“… followed by Cluster 1 (14% of the total) and Cluster 2 (3% of the total) (see supplementary tables 5 and 6 for detailed results and analyzed sequences).”<br><br><br>The due date for submitting the revised version of your article is 15 Oct 2018.<br><br>We look forward to receiving your revised manuscript soon.<br><br>Best wishes,<br><br>Nicole Nogoy, Ph.D<br>GigaScience |
| **Additional Information:** | |

*Powered by Editorial Manager® and ProduXion Manager® from Aries Systems Corporation*

| Question | Response |
| --- | --- |
| Are you submitting this manuscript to a special series or article collection? | No |
| **Experimental design and statistics**<br><br>Full details of the experimental design and statistical methods used should be given in the Methods section, as detailed in our Minimum Standards Reporting Checklist. Information essential to interpreting the data presented should be made available in the figure legends.<br><br>Have you included all the information requested in your manuscript? | Yes |
| **Resources**<br><br>A description of all resources used, including antibodies, cell lines, animals and software tools, with enough information to allow them to be uniquely identified, should be included in the Methods section. Authors are strongly encouraged to cite Research Resource Identifiers (RRIDs) for antibodies, model organisms and tools, where possible.<br><br>Have you included the information requested as detailed in our Minimum Standards Reporting Checklist? | Yes |
| **Availability of data and materials**<br><br>All datasets and code on which the conclusions of the paper rely must be either included in your submission or deposited in publicly available repositories (where available and ethically appropriate), referencing such data using a unique identifier in the references and in the "Availability of Data and Materials" section of your manuscript. | Yes |

*Powered by Editorial Manager® and ProduXion Manager® from Aries Systems Corporation*

| | |
|---|---|
| Have you have met the above requirement as detailed in our Minimum Standards Reporting Checklist? | |

Powered by Editorial Manager® and ProduXion Manager® from Aries Systems Corporation

Manuscript with DOI

Click here to access/download;Manuscript;NAT-NCS2-hound_revised_20181011_NO_track_changes copy w DOI.docx

Click here to view linked References

**TITLE: NAT/NCS2-hound: A webserver for the detection and evolutionary classification of prokaryotic and eukaryotic nucleobase-cation symporters of the NAT/NCS2 family.**

Chaliotis A[1a], Vlastaridis P[1a], Ntountoumi, C[1a], Botou M[2], Yalelis V[2], Lazou P[2], Tatsaki E[2], Mossialos D[3], Frillingos S[2*] and Amoutzias GD[1*]

[1]Bioinformatics Laboratory, Department of Biochemistry and Biotechnology, University of Thessaly, Larisa, 41500, Greece

[2]Laboratory of Biological Chemistry, Department of Medicine, University of Ioannina, Ioannina, Greece.

[3]Molecular Bacteriology Laboratory, Department of Biochemistry and Biotechnology, University of Thessaly, Larisa, 41500, Greece

Emails: anargyros.chaliotis@gmail.com, panosvlastaridis@gmail.com, crisdudu1394@yahoo.com, mpotoumaria@yahoo.gr, v-yyial@hotmail.com, lazoup@gmail.com, ctatsaki@yahoo.gr, mosial@bio.uth.gr, efriligo@uoi.gr, amoutzias@bio.uth.gr

Keywords: Nucleobase transporters, nucleobase-ascorbate transporter (NAT) family, nucleobase-cation symporter-2 (NCS2) family, prokaryotes, Evolution, MEME motifs

[a]These authors contributed equally.

[*]To whom correspondence should be addressed: Grigoris D. Amoutzias or Stathis Frillingos.

# Abstract

Nucleobase transporters are important for supplying the cell with purines and/or pyrimidines, for controlling the intracellular pool of nucleotides and for obtaining exogenous nitrogen/carbon sources for the metabolism. Nucleobase transporters are also evaluated as potential targets for antimicrobial therapies, since several pathogenic microorganisms rely on purine/pyrimidine salvage from their hosts. The majority of known nucleobase transporters belong to the evolutionarily conserved and ubiquitous NAT/NCS2 protein family. Based on a large-scale phylogenetic analysis that we performed on thousands of prokaryotic proteomes, we have developed a webserver that can detect and distinguish this family of transporters from other homologous families that recognize different substrates. We can further categorize these transporters to certain evolutionary groups with distinct substrate preferences. The webserver scans whole proteomes and graphically displays which proteins are identified as NAT/NCS2, to which evolutionary groups and subgroups they belong to and which conserved motifs they have. For key subgroups and motifs, the server displays annotated information from published crystal-structures and mutational studies pointing to key functional amino acids that may help experts assess the transport capability of the target sequences. The server is 100% accurate in detecting NAT/NCS2 family members. We also used the server to analyze

9109 prokaryotic proteomes and identified Clostridia, Bacilli, β- and γ-Proteobacteria, Actinobacteria and Fusobacteria as the taxa with the largest number of NAT/NCS2 transporters per proteome. An analysis of 120 representative eukaryotic proteomes also demonstrates the server's capability of correctly analyzing this major lineage, with plants emerging as the group with the highest number of NAT/NCS2 members per proteome.

# Introduction

The NAT/NCS2 (Nucleobase-Ascorbate Transporter / Nucleobase-Cation Symporter-2) protein family encompasses ion-gradient driven transporters of key metabolites or anti-metabolite analogs with diverse substrate preferences, ranging from purine or pyrimidine permeases in various organisms to $Na^+$-dependent vitamin C transporters in human and other mammals [1–6]. Their additional function as providers of nitrogen/carbon source may also affect energy production, replication and protein synthesis through the salvage pathways for nucleotide synthesis [7–9]. In addition to their important direct role on the central metabolism of the cell, these and other nucleobase transporters have attracted interest as potential targets of purine/pyrimidine-based antimicrobials that could either be selectively routed into target cells to act as anti-metabolites or selectively inhibit an essential nucleobase transporter of the target cell [10–14]

This protein family is one of the 18 known families of the APC superfamily [15] and represents a subset of APC families which conform to a distinct structural/mechanistic pattern. The NAT/NCS2 transporters consist of 14 transmembrane segments (TMs) divided in two inverted repeats (7+7) and arranged spatially into a core domain (TMs 1-4 and 8-11) and a gate domain (TMs 5-7 and 12-14) [16]. The core domain contains all major determinants of the substrate-binding site, whereas the gate domain contributes to alternating access by allowing conformational rearrangements and providing major gating elements. The proteins probably function as homodimers and may use an elevator-like mechanism to achieve alternating access [17,18]. Similar structural features are described for transporters of two other APC families, the Sulfate Permeases (SulP) [19] and the Anion Exchangers (AE) which includes the well studied chloride/bicarbonate exchanger (band 3) of human erythrocytes [20].

The NAT/NCS2 is split phylogenetically in two subfamilies. The first one, COG2233 or NAT, contains bacterial and fungal permeases for purines (xanthine, uric acid), bacterial permeases for pyrimidines (uracil, thymine), plantal and mammalian broad-specificity uracil/purine permeases (not present in human), and the mammalian L-ascorbate transporters SVCT1 and SVCT2. Insight on the transport mechanism of this subfamily has been provided by high-resolution crystal structures for two members, the uracil permease UraA of *E. coli* [16,18] and the xanthine/uric acid permease UapA of *Aspergillus nidulans* [17], coupled with extensive mutagenesis studies on UapA [21], the xanthine permease XanQ of *E. coli* [1,22] and few other homologs [23,24]. The other subfamily, COG2252 or AzgA-like [25], contains bacterial, fungal and plantal permeases for salvageable purines (adenine, guanine, hypoxanthine) which are less well studied with respect to structure-function relationships [7,26].

Despite their importance, membrane transporters in general and the NAT/NCS2 family in particular are not so extensively studied to date as other categories of proteins are, due to the inherent difficulties in experimentation and in accurate prediction of their function [27,28]. Based on a large-scale evolutionary analysis that we performed in this study, we have i) identified in prokaryotes the major evolutionary groups and subgroups, with distinct substrate specificities, ii) identified key motifs for each phylogenetic group and subgroup that are related to substrate specificity, iii) developed a webserver that utilizes all the above information to detect and classify at proteome-scale NAT/NCS2 transporters and iv) analyzed with this webserver 9109 prokaryotic and 120 Eukaryotic proteomes so as to investigate which evolutionary lineages are rich in these transporters. We expect that this type of analyses and the accompanying computational tool, which are lacking in general for other families of transporters, will facilitate the experimental study of new homologs, provide a practical tool for assignment of homologs into functionally-relevant associated subgroups and also improve their annotation in the databases.

# Materials and Methods

**Development of HMMs and Meme motifs for the family, subfamilies and evolutionary clusters.**

All the annotated sequences of the 2A APC superfamily (organized in 18 families) were obtained from TCDB [15]. For each of the 18 families we generated protein alignments with Muscle and Seaview [29,30] that were manually edited and then used to build a hidden markov model for each one of them with HMMER [31].

Next, 4442 Bacterial AND 213 Archaeal Proteomes were downloaded from UNIPROT (January 2017) [32]. Their protein sequences were scanned with the above 18 HMMs and thus, 8291 proteins of the NAT/NCS2 family were identified and retained for further analysis. Afterwards, close homologs were removed with the Blastclust software, using as cutoff 70% protein identity over 70% of sequence length. Thus 1355 NAT/NCS2 sequences were retained after this step.

Subsequently, these sequences were fed to the MEME software [33] so as to identify 14 motifs of length 14-21 or 18-25 amino acids each. Manual inspection of sequences with a very low number of motifs resulted in rejection of 14 sequences. Thus 1341 sequences were retained. These 1341 sequences were scanned again with the 14 MEME motifs, by MAST [33]. Custom Perl scripts were developed to obtain the motif presence/absence for each sequence as a vector of 0 and 1 values, based on detection with MAST (see supplementary folder Custom_scipts). The above vectors were clustered in MATLAB with the Clustergram function (default parameters – commands found in supplementary folder Custom_scipts). This first round of clustering revealed two major evolutionary subfamilies, designated SF1 and SF2 (see supplementary figure S1). The sequences of each subfamily were fed to another round of MEME motif detection with the same parameters as in the first instance. Again, 14 MEME-motifs were made for each

subfamily. These were used with the MAST software to identify MEME-Motif content for each subfamily, and again, vectors of motif presence/absence were generated for each subfamily (and their clusters were manually inspected; see supplementary figures S2 and S3). All Meme/Mast results and analyzed sequences are found in the supplementary folder “MEME_MAST_motifs”.

Afterwards, the protein sequences of each subfamily separately were aligned and manually edited with Muscle and Seaview [29,30]. Furthermore, in each subfamily, sequences with experimental evidence of substrate specificity were added (eukaryotic ones as well). Phylogenetic trees were generated with the BioNJ method using the Poisson model and 1000 bootstraps. The two generated phylogenetic trees (for each of the two distinct subfamilies – see supplementary figures S4 and S5) were annotated and visualized in Archaeopteryx and Treedyn [34,35]. Subfamily 1 was organized in six major and four very small clusters. Subfamily 2, that was more homogeneous than subfamily 1, was organized in many small clusters. Hidden Markov Models were thus constructed for the NAT/NCS2 family, its two subfamilies and for each of the 6 major clusters in subfamily 1. For several of the small clusters in subfamily 2 that contained sequences with known substrates we also generated extra HMMs. In addition, we generated 14 MEME motifs for each subfamily and each of the 6 clusters in subfamily 1. A workflow of how the various HMMs and MEME motifs were generated is found in supplementary figure S12_workflow_diagram. All edited sequence alignments, HMMs and phylogenetic trees (in newick format) are organized in supplementary folder “Sequence_alignments_HMMs_phylogenetic_trees”.

**Development and Evaluation of the server**

All the above HMMs and MEME motifs were incorporated in a webserver, named NAT/NCS2-hound, that may scan protein sequences in FASTA format, identify members of this family and further classify them in the various subfamilies and clusters. The webserver is based on the Jhipster Application Framework [36] that utilizes Angular Javascript Framework for the front-end and the Java language and Spring Framework for the back-end. The server is freely available at [37].

The server and instructions for local installation are found in supplementary folder “Server_for_local_installation”. Also, the server is registered at SciCrunch.org with RRID: SCR_016473.

Functional information for the various amino acids was obtained from several mutational studies [1,21,24] and from the structural studies on UraA [16,18] and UapA [17].

We performed an evaluation analysis, in order to assess the effectiveness of the NAT/NCS2-hound server. TCDB annotated transporters of the 18 families of the APC superfamily were used as bait to obtain best blast hits against bacterial reference proteomes downloaded from Uniprot. The best blast hit of a bait sequence was designated as a member of the family that its annotated (from TCDB) bait sequence belonged to. These retrieved best blast hit sequences constituted the evaluation set. Any of these sequences that had been used to train the HMMs were removed from the evaluation set.

Thus, we retrieved/retained 7799 APC sequences, of which 975 belonged to the NAT/NCS2 family. These were scanned by our server for detection and evolutionary classification. The server demonstrated 100% accuracy (100% sensitivity and 100% specificity) in detecting NAT/NCS2 family members and can further categorize them to the various evolutionary subgroups, display conserved motifs and relevant functional information/annotation.

In order to assess the distribution of NAT/NCS2 family in major taxonomic lineages, 9109 prokaryotic proteomes (downloaded from NCBI at March 2018) and 120 Eukaryotic Reference Proteomes (downloaded from Uniprot at March 2018) were scanned by our server. The presence of a minimum number of seven MEME motifs was required as a cutoff, to filter out any sequence fragments. All results and sequence IDs are found in supplementary tables 1-6.

# Results and Discussion

**The NAT/NCS2 family is organized in two major subfamilies.**
An analysis of 1341 proteins, based on the presence/absence of conserved MEME motifs within the NAT/NCS2 family clearly revealed the presence of two distinct and major subfamilies (see supplementary figure S1). Previous phylogenetic analyses also revealed the presence of these two major subfamilies [7], in accordance with the presence of two COGs, designated as COG2233 (Xanthine/Uracil permease) and COG2252 (AzgA-like). Subfamily 1 (COG2233) consisted of 748 sequences and subfamily 2 (COG2252, AzgA-like) consisted of 593 sequences. The members of Subfamily 1 display a greater degree of sequence divergence among them, whereas members of subfamily 2 constitute a more homogeneous set of sequences (Supplementary figures S2-S3).

**Distinct Phylogenetic clusters within Subfamily 1.**
Further phylogenetic analysis of the more diverse members of Subfamily 1 reveals the clear presence of six major clusters (Clusters 1-6) and four minor clusters (see Figure 1). The incorporation of functionally annotated sequences from all kingdoms of life further helped us understand the substrate specificity profile of each cluster, whenever relevant information for representative homologs was available. The largest and most diverse cluster (Cluster 1) contains sequences that have been annotated to transport Xanthine or Uric Acid or both. The second largest cluster (Cluster 2) contains sequences that are known to transport Uracil or Uracil and Thymine. The third largest cluster (Cluster 3) contains the YbbY gene from *E.coli*, a homolog that is not functionally annotated in the databases but recent evidence suggests that it transports adenine, guanine and hypoxanthine (Botou and Frillingos, unpublished data). The fourth cluster (Cluster 4) contains functionally characterized sequences from Eukaryotes, but also encompasses functionally unknown homologs from Archaea as well as a few Bacteria. All the other clusters do not contain any sequences of known function.

Subfamily 2 is more homogeneous and is organized in many small clusters, with small differences among them. For several of those small clusters that contain sequences with known substrates we generated additional HMMs. A more detailed inspection of the various phylogenetic trees for each subfamily and each of the major 6 clusters within subfamily 1 are available in supplementary materials (Supplementary figures S4-S11).

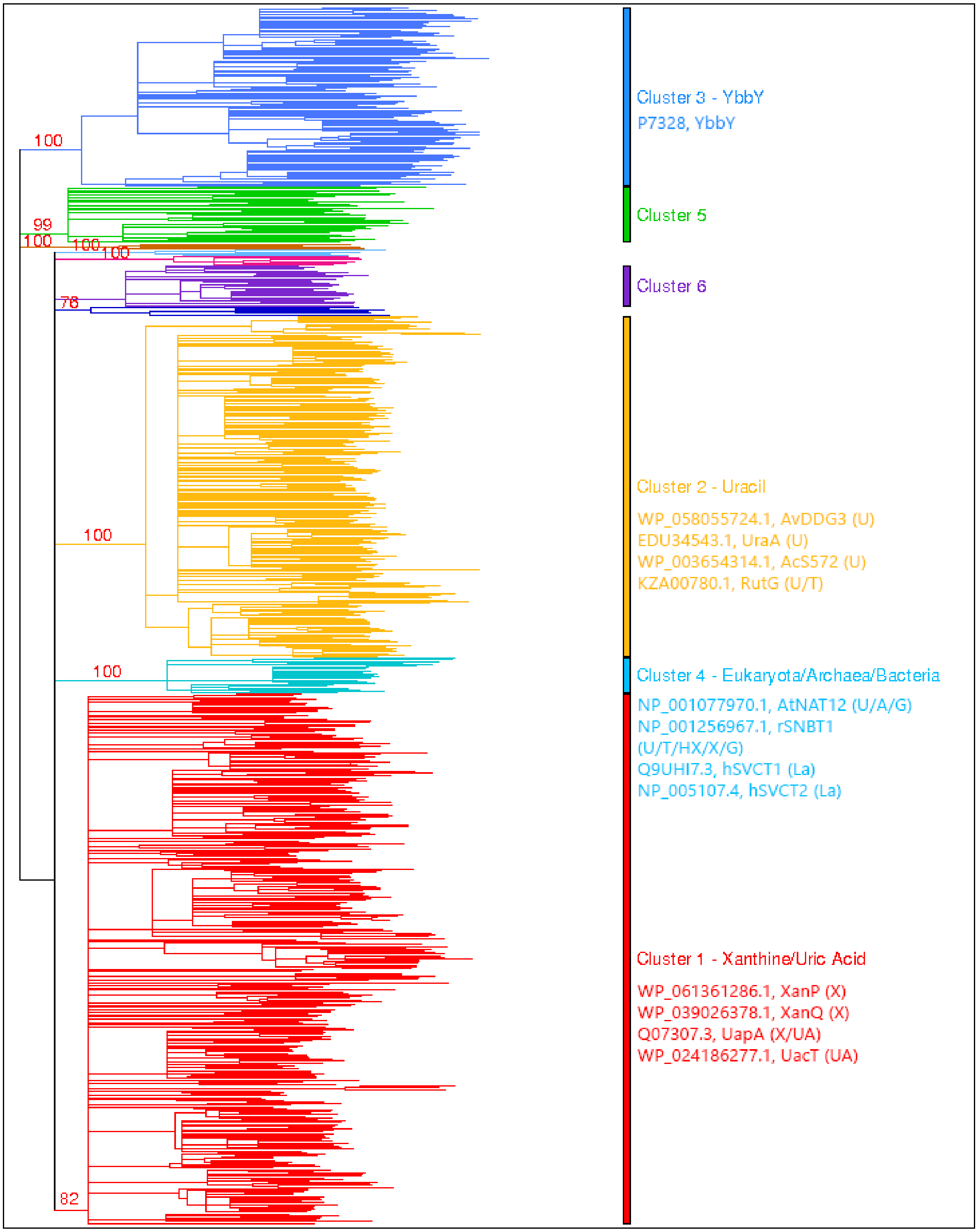

Figure 1. Phylogenetic tree of Subfamily 1 of the NAT/NCS2 family. The various phylogenetic clusters are depicted with different colors. Sequence redundancy was removed at a cutoff of 70% protein identity over 70% of sequence length. Well characterized known homologs are indicated with their major substrates in parenthesis (U, uracil; T, thymine; A, adenine; G, guanine; HX, hypoxanthine; La, L-ascorbic acid; X, xanthine, UA, uric acid).

**A web server for the detection and evolutionary classification of NAT/NCS2 family members.**

All the above evolutionary analyses, the HMMs and MEME motifs generated for the various subfamilies and evolutionary clusters have been used to develop a web server for the detection and evolutionary classification of NAT/NCS2 family members, named NAT/NCS2-hound. The webserver is freely available at [37]..This webserver detects and distinguishes this family of transporters from the other 17 homologous families of the APC superfamily. Furthermore, it can categorize these transporters to certain subfamilies and clusters associated with distinct substrate specificities, based on the large-scale phylogenetic analysis that we performed on prokaryotic proteomes. For each one of them separately, the identified set of characteristic signature motifs is detected. Furthermore, for several key subgroups we have integrated information from published crystal-structures and mutational studies to help experts identify key functional amino acids and help them assess the transport capability of the scanned sequences. Nevertheless, this server does not function as a prediction tool of substrate specificity. The NAT/NCS2-hound server implements for this important family the same principles and computational protocol that were developed/implemented recently for another prokaryotic superfamily, the tRNA-synthetases [38].

The input for this server is a protein sequence or a proteome file in FASTA format. The webserver displays graphically (see figure 2) which proteins have been identified as NAT/NCS2, to which subfamily and cluster they belong to and which conserved motifs have been identified on the target proteins. For several key subgroups and motifs, the server further displays annotated (by our experts) information from published crystal-structures and mutational studies pointing to key functional amino acids of well-studied representative homologs.

The server has been evaluated against a dataset of 7800 homologous transporters of the APC superfamily, of which 975 belong to the NAT/NCS2 family and displayed 100% accuracy (100% sensitivity and 100% specificity).

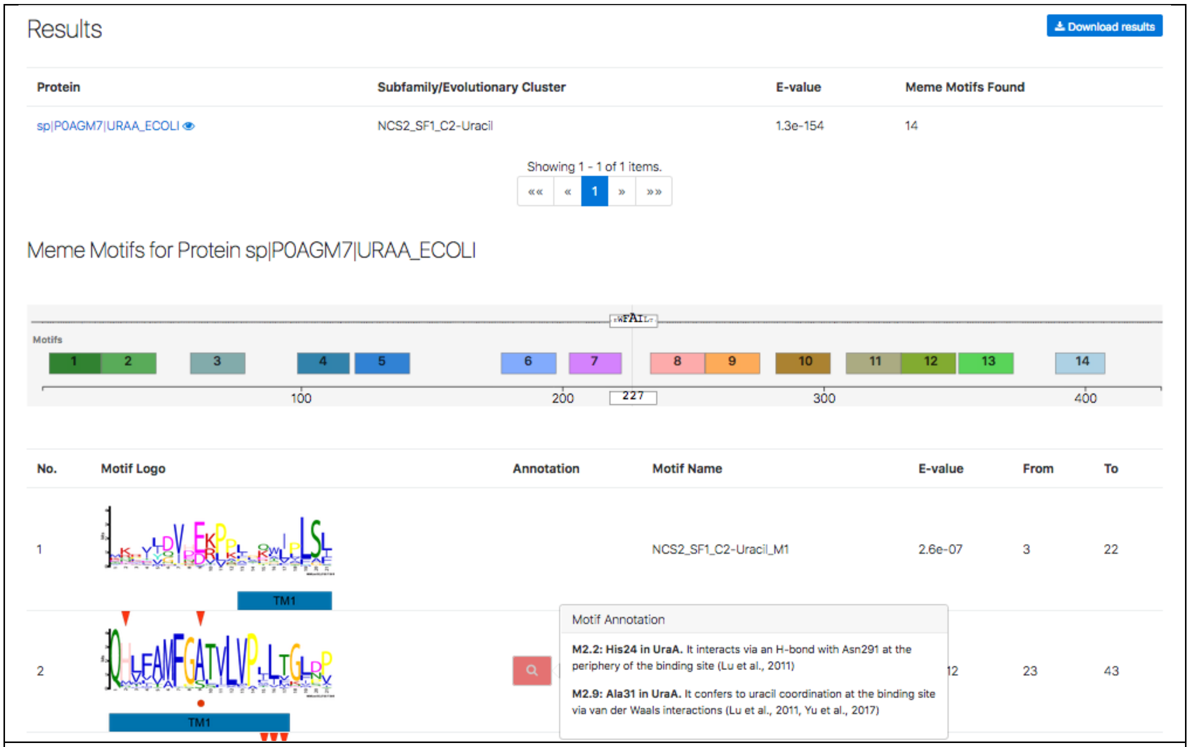


Figure 2. Display of results from the NAT/NCS2-hound server, including the best Hidden Markov Model that detects the protein sequence, the various MEME conserved motifs and any available functional information/annotation for specific sites in certain motifs.

**Distribution of the NAT/NCS2 family and subfamilies in Prokaryotes and Eukaryotes.**

In order to assess the distribution of NAT/NCS2 members in major taxonomic lineages, we scanned 9109 prokaryotic proteomes (from NCBI) and 120 representative Eukaryotic Proteomes (from Uniprot). As a filter, we only included in our analysis 29096 prokaryotic and 361 eukaryotic NAT/NCS2 proteins that had at least 7 Meme motifs each, to exclude small sequence fragments.

80% (7318/9109) of the prokaryotic proteomes had at least one NAT/NCS2 protein, based on our criteria, with 4 NAT/NCS2 proteins, on average. The lineages with the most proteins (on average) are Clostridia, Bacilli, β- and γ-Proteobacteria and Actinobacteria (see figure 3). The proteomes with the most transporters were *Clostridium bolteae* (with 14 members), several different γ-Proteobacteria (*Morganella morganii*, *Enterobacter lignolyticus*, *Citrobacter amalonaticus*) and *Bacillus megaterium* with 11 members each, followed by many other γ-Proteobacteria (such as *E.coli*) with 10 members each (see supplementary tables 1 & 2). Subfamily 1 was more abundant than subfamily 2, comprising of 58% of all NAT/NCS2 proteins (paired t-test p-value =0). Also, Cluster 1 (Xanthine/Uric acid) and Cluster 2 (Uracil) from subfamily 1, comprised of 29% and 23% of the total number of NAT/NCS2 proteins detected, whereas Cluster 3 (YbbY) was the third largest one, comprising of 5% of the total number of NAT/NCS2 proteins.

637 of the prokaryotic species included in this analysis were represented by at least two strains each, of which 97 species had 10-50 strains, 10 species with 50-100 strains and 9

species had over 100 strains (see supplementary_table_3_strain_volatility.csv). For 73% (463/637) of these studied species there was no change in the number of NAT/NCS2 proteins among the various strains of a certain species. Striking examples are 4 species with more than 100 strains each (*Staphylococcus aureus*, *Listeria monocytogenes*, *Mycobacterium tuberculosis*, *Campylobacter jejuni*) that displayed no difference in the numbers of NAT/NCS2 proteins among different strains. On the other end, the most notable exceptions were *Streptomyces hygroscopicus* (4 strains, with 3-9 homologs per strain), *Clostridium botulinum* (34 strains, 5-10 homologs), *Pseudomonas fluorescens* (16 strains, 4-8 homologs) and *Pseudomonas stutzeri* (10 strains, 3-7 homologs).

The number of NAT/NCS2 proteins per strain ranged from 14 to 0 (see supplementary tables 2 & 3). Most strains that contain no NAT/NCS2 homologs belong to parasitic and/or endosymbiotic bacteria (*Chlamydia*, *Rickettsia*, *Ehrlichia*, *Wolbachia*, *Coxiella*, *Leptospira*, *Capnocytophaga*, *Cellulophaga*, *Bartonella*, *Mycoplasma*, *Mycobacterium* (17/40 species), *Flavobacterium* (4/8 species), *Xanthomonas oryzae*, *Xylella fastidiosa*, *Mesorhizobium* (5/6 species), *Rhizobium* (4/5 species), *Liberibacter*, *Buchnera aphidicola*) or autotrophic bacteria or archaea with specialized metabolic adaptations (*Sulfolobus*, *Metallosphaera*, *Aquifex*, *Sulfurimonas*, *Halothiobacillus*, *Dehalococcoides*, *Oligotropha*, *Methanosarcina* (7/8 species), *Methanobacterium* (4/5 species), *Prochlorococcus*, *Rhodobacter*, *Rhodopseudomonas*). It is of interest that some known pathogenic bacteria which rely on purine salvage for nucleotide biosynthesis and infectivity [13,39–41] retain few NAT/NCS2 homologs all of which belong to subfamily 2 (classified as adenine/guanine/hypoxanthine transporters) (*Helicobacter pylori* (85/85 strains, 1 homolog), *Borrelia burgdorferi* (8/9 strains, 2 homologs)). On the other end, 2644 strains (29% of the total) which are distributed in 546 species (17% of the total) had 5 or more NAT/NCS2 homologs, with an average of 6 homologs per species (3.5-3.7 in subfamily 1 and 2.3-2.4 in subfamily 2). All these strains are Bacterial and most belong to heterotrophic, metabolically versatile species; their NAT/NCS2 homologs are distributed in several phylogenetic clusters indicating a range of different nucleobase preferences. As an example, *Escherichia coli* K-12 which has been studied thoroughly with respect to the relevant functional profiles contains 10 NAT/NCS2 proteins, including the xanthine-specific transporters XanP and XanQ [42] and the uric acid transporter UacT [23] in Cluster 1 (see figure 1), the uracil-specific UraA and the broader-specificity uracil/thymine transporter RutG [24] in Cluster 4, the purine transporter YbbY in Cluster 3, and two pairs of closely related transporters specific for adenine (AdeP, AdeQ) or guanine and hypoxanthine (GhxP, GhxQ) [7] that belong to Subfamily 2. This versatility in substrate profiles is associated with different metabolic pathways and linkage of the transporter genes with different catabolic or biosynthetic operons [1,24].

Furthermore, we did a survey on 61 proteomes from strains found in the human microbiome [43] and found that NAT/NCS2 homologs are enriched (3 per genome, on average) in bacteria of the gastrointestinal tract (see supplementary table 4).

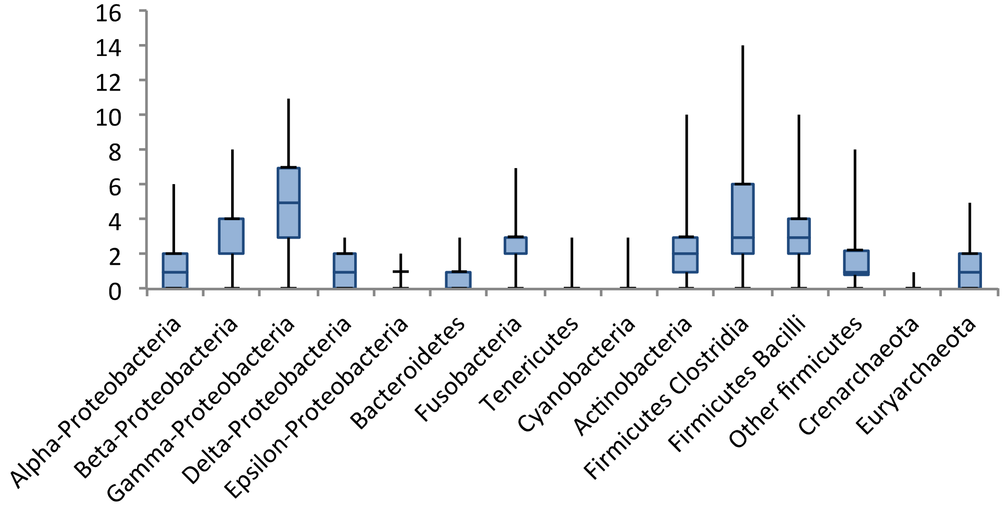


Figure 3. Boxplot of the distribution of NAT/NCS2 proteins per proteome, in major prokaryotic taxonomic lineages. The analysis was based on 9109 Bacterial and Archaeal proteomes. β and γ-Proteobacteria, Clostridia, Bacilli, Fusobacteria and Actinobacteria had the highest numbers of transporters per proteome (on average).

We also analyzed 120 representative proteomes from animals, fungi, plants and various unicellular Eukaryotes. 83% of the detected proteins were found to belong to subfamily 1 (the rest in subfamily 2), with the vast majority of them (66% of the total) in Cluster 4, followed by Cluster 1 (14% of the total) and Cluster 2 (3% of the total) (see supplementary tables 5 and 6 for detailed results and analyzed sequences). As an additional validation step, our server detected and properly classified well-known and previously annotated NAT/NCS2 sequences in certain selected species, such as the human homologs SVCT1 and SVCT2 [5], the rat uracil/purine transporter rSNBT1 [4], the uracil/purine transporters AtNAT3 and AtNAT12 and adenine/guanine transporters AtAzg1 and AtAzg2 of *Arabidopsis thaliana* [3], and the xanthine/uric acid transporters UapA and UapC and adenine/guanine/hypoxanthine transporter AzgA of *Aspergillus nidulans* [2]. Thus, although the development of the server was based on prokaryotic sequences, the server can successfully analyze eukaryotic sequences as well. This is attributed to the fact that all eukaryotic sequences are fully contained within evolutionary groups that are already present in Prokaryotes. Plants had the highest number of NAT/NCS2 members (10 per genome on average), followed by animals (3 on average), then by fungi (2 on average), whereas most (73%) of the unicellular eukaryotes had no NAT/NCS2 proteins. Intriguingly, Metazoa had only sequences that belonged to Cluster 4 (of subfamily 1). Plants also displayed a great expansion in members of Cluster 4, but they also had small numbers of sequences from Cluster 1 and subfamily 2 (AzgA-like). Fungi had a rather balanced number of sequences from Cluster 1 and subfamily 2. Notably, Tobacco (*Nicotiana tabacum)* had the most (30) NAT/NCS2 proteins, the fungus *Basidiobolus meristosporus* had 16 members, whereas two lophotrochozoa (*Lingula unguis and Crassostrea gigas*) had 12 members each. Although it is conceivable that the percentages observed in this analysis may change depending on the species

sampling, the general trends observed for each major taxonomic lineage are expected to hold.

## Conclusions

Based on a large-scale phylogenetic analysis of prokaryotic NAT/NCS2 proteins, a webserver has been developed that may scan whole proteomes and identify members of this family. The server classifies these members in various subfamilies and evolutionary clusters with certain substrate profiles and identifies conserved motifs that are related to function. An analysis of 9109 prokaryotic proteomes with our server revealed that the evolutionary lineages containing the largest numbers of NAT/NCS2 members are β- and γ-Proteobacteria, Bacilli, Clostridia, Actinobacteria and Fusobacteria. An analysis of 120 Eukaryotic proteomes also revealed that this server is fully capable of successfully analyzing this taxonomic lineage as well.

## Availability of supporting source code and requirements

Project name: NAT-NCS2
Project home page: https://github.com/pvlastaridis/nat-ncs2
Operating system(s): Platform independent
Programming language: Java, Angular, Spring
Other requirements: Java JDK8, NodeJS 8.10, Yarn(yarnpkg.com) 1.10, Hmmer(http://hmmer.org/) 3.1
License: MIT
RRID: SCR_016473

## Availability of supporting data

All supplementary data can be downloaded from the NAT/NCS2-hound server at: http://bioinf.bio.uth.gr/nat-ncs2/. Further supporting data and snapshots of the code are openly available in the *GigaScience* repository, GigaDB [44].

## Author's Contributions

AC and CN performed the phylogenetic analyses, PV developed the server, MB, VY, PL, ET gathered annotation and functional information. DM, SF and GDA conceived the study, supervised the students and prepared the manuscript.

## Funding

G.D.A acknowledges support from the "ARISTEIA II" Action of the "OPERATIONAL PROGRAMME EDUCATION AND LIFELONG LEARNING" that is co-funded by the European Social Fund (ESF) and National Resources [grant number 4288 to G.D.A]. G.D.A. also acknowledges support by research grants from the Postgraduate Program 'Applications of Molecular Biology-Genetics, Diagnostic Biomarkers', [code 3817] of the University of Thessaly, School of Health Sciences, Department of Biochemistry & Biotechnology.

## Competing interests

The authors declare that they have no competing interests.

## References


1. Frillingos S. Insights to the evolution of Nucleobase-Ascorbate Transporters (NAT/NCS2 family) from the Cys-scanning analysis of xanthine permease XanQ. Int J Biochem Mol Biol. 2012;3:250–72.

2. Gournas C, Papageorgiou I, Diallinas G. The nucleobase-ascorbate transporter (NAT) family: genomics, evolution, structure-function relationships and physiological role. Mol Biosyst. 2008;4:404–16.

3. Girke C, Daumann M, Niopek-Witz S, Möhlmann T. Nucleobase and nucleoside transport and integration into plant metabolism. Front Plant Sci. 2014;5:443.

4. Yamamoto S, Inoue K, Murata T, Kamigaso S, Yasujima T, Maeda J, et al. Identification and functional characterization of the first nucleobase transporter in mammals: implication in the species difference in the intestinal absorption mechanism of nucleobases and their analogs between higher primates and other mammals. J Biol Chem. 2010;285:6522–31.

5. Bürzle M, Suzuki Y, Ackermann D, Miyazaki H, Maeda N, Clémençon B, et al. The sodium-dependent ascorbic acid transporter family SLC23. Mol Aspects Med. 2013;34:436–54.

6. Kourkoulou A, Pittis AA, Diallinas G. Evolution of substrate specificity in the Nucleobase-Ascorbate Transporter (NAT) protein family. Microb Cell. 2018;5:280–92.

7. Papakostas K, Botou M, Frillingos S. Functional identification of the hypoxanthine/guanine transporters YjcD and YgfQ and the adenine transporters PurP and YicO of Escherichia coli K-12. J Biol Chem. 2013;288:36827–40.

8. King AE, Ackley MA, Cass CE, Young JD, Baldwin SA. Nucleoside transporters: from scavengers to novel therapeutic targets. Trends Pharmacol Sci. 2006;27:416–25.

9. Kozmin SG, Stepchenkova EI, Chow SC, Schaaper RM. A critical role for the putative NCS2 nucleobase permease YjcD in the sensitivity of Escherichia coli to cytotoxic and mutagenic purine analogs. MBio. 2013;4:e00661-00613.

10. Köse M, Schiedel AC. Nucleoside/nucleobase transporters: drug targets of the future? Future Med Chem. 2009;1:303–26.

11. Landfear SM. Transporters for drug delivery and as drug targets in parasitic protozoa. Clin Pharmacol Ther. 2010;87:122–5.

12. Ferrari V, Serpi M. Nucleoside analogs and tuberculosis: new weapons against an old enemy. Future Med Chem. 2015;7:291–314.

13. Jain S, Showman AC, Jewett MW. Molecular dissection of a Borrelia burgdorferi in vivo essential purine transport system. Infect Immun. 2015;83:2224–33.

14. Lougiakis N, Gavriil E-S, Kairis M, Sioupouli G, Lambrinidis G, Benaki D, et al. Design and synthesis of purine analogues as highly specific ligands for FcyB, a ubiquitous fungal nucleobase transporter. Bioorg Med Chem. 2016;24:5941–52.

15. Saier MH, Reddy VS, Tsu BV, Ahmed MS, Li C, Moreno-Hagelsieb G. The Transporter Classification Database (TCDB): recent advances. Nucleic Acids Res. 2016;44:D372-379.

16. Lu F, Li S, Jiang Y, Jiang J, Fan H, Lu G, et al. Structure and mechanism of the uracil transporter UraA. Nature. 2011;472:243–6.

17. Alguel Y, Amillis S, Leung J, Lambrinidis G, Capaldi S, Scull NJ, et al. Structure of eukaryotic purine/H(+) symporter UapA suggests a role for homodimerization in transport activity. Nat Commun. 2016;7:11336.

18. Yu X, Yang G, Yan C, Baylon JL, Jiang J, Fan H, et al. Dimeric structure of the uracil:proton symporter UraA provides mechanistic insights into the SLC4/23/26 transporters. Cell Res. 2017;27:1020–33.

19. Geertsma ER, Chang Y-N, Shaik FR, Neldner Y, Pardon E, Steyaert J, et al. Structure of a prokaryotic fumarate transporter reveals the architecture of the SLC26 family. Nat Struct Mol Biol. 2015;22:803–8.

20. Arakawa T, Kobayashi-Yurugi T, Alguel Y, Iwanari H, Hatae H, Iwata M, et al. Crystal structure of the anion exchanger domain of human erythrocyte band 3. Science. 2015;350:680–4.

21. Diallinas G. Dissection of Transporter Function: From Genetics to Structure. Trends Genet. 2016;32:576–90.

22. Karena E, Tatsaki E, Lambrinidis G, Mikros E, Frillingos S. Analysis of conserved NCS2 motifs in the Escherichia coli xanthine permease XanQ. Mol Microbiol. 2015;98:502–17.

23. Papakostas K, Frillingos S. Substrate selectivity of YgfU, a uric acid transporter from Escherichia coli. J Biol Chem. 2012;287:15684–95.

24. Botou M, Lazou P, Papakostas K, Lambrinidis G, Evangelidis T, Mikros E, et al. Insight on specificity of uracil permeases of the NAT/NCS2 family from analysis of the transporter encoded in the pyrimidine utilization operon of Escherichia coli. Mol Microbiol. 2018;108:204–19.

25. Cecchetto G, Amillis S, Diallinas G, Scazzocchio C, Drevet C. The AzgA purine transporter of Aspergillus nidulans. Characterization of a protein belonging to a new phylogenetic cluster. J Biol Chem. 2004;279:3132–41.

26. Krypotou E, Lambrinidis G, Evangelidis T, Mikros E, Diallinas G. Modelling, substrate docking and mutational analysis identify residues essential for function and specificity of the major fungal purine transporter AzgA. Mol Microbiol. 2014;93:129–45.

27. César-Razquin A, Snijder B, Frappier-Brinton T, Isserlin R, Gyimesi G, Bai X, et al. A Call for Systematic Research on Solute Carriers. Cell. 2015;162:478–87.

28. Elbourne LDH, Tetu SG, Hassan KA, Paulsen IT. TransportDB 2.0: a database for exploring membrane transporters in sequenced genomes from all domains of life. Nucleic Acids Res. 2017;45:D320–4.

29. Gouy M, Guindon S, Gascuel O. SeaView version 4: A multiplatform graphical user interface for sequence alignment and phylogenetic tree building. Mol Biol Evol. 2010;27:221–4.

30. Edgar RC. MUSCLE: multiple sequence alignment with high accuracy and high throughput. Nucleic Acids Res. 2004;32:1792–7.

31. Eddy SR. Accelerated Profile HMM Searches. PLoS Comput Biol. 2011;7:e1002195.

32. UniProt Consortium. UniProt: a hub for protein information. Nucleic Acids Res. 2015;43:D204-212.

33. Bailey TL, Johnson J, Grant CE, Noble WS. The MEME Suite. Nucleic Acids Res. 2015;43:W39-49.

34. Han MV, Zmasek CM. phyloXML: XML for evolutionary biology and comparative genomics. BMC Bioinformatics. 2009;10:356.

35. Chevenet F, Brun C, Bañuls A-L, Jacq B, Christen R. TreeDyn: towards dynamic graphics and annotations for analyses of trees. BMC Bioinformatics. 2006;7:439.

36. JHipster - Generate your Spring Boot + Angular/React applications! [Internet]. [cited 2018 Oct 9]. Available from: https://www.jhipster.tech/

37. Prediction and Evolutionary Classification Server of prokaryotic and eukaryotic NAT/NCS2 transporters [Internet]. [cited 2018 Oct 9]. Available from: http://bioinf.bio.uth.gr/nat-ncs2/

38. Chaliotis A, Vlastaridis P, Mossialos D, Ibba M, Becker HD, Stathopoulos C, et al. The complex evolutionary history of aminoacyl-tRNA synthetases. Nucleic Acids Res. 2017;45:1059–68.

39. Jain S, Sutchu S, Rosa PA, Byram R, Jewett MW. Borrelia burgdorferi harbors a transport system essential for purine salvage and mammalian infection. Infect Immun. 2012;80:3086–93.

40. Liechti G, Goldberg JB. Helicobacter pylori relies primarily on the purine salvage pathway for purine nucleotide biosynthesis. J Bacteriol. 2012;194:839–54.

41. Liechti GW, Goldberg JB. Helicobacter pylori salvages purines from extracellular host cell DNA utilizing the outer membrane-associated nuclease NucT. J Bacteriol. 2013;195:4387–98.

42. Karatza P, Frillingos S. Cloning and functional characterization of two bacterial members of the NAT/NCS2 family in Escherichia coli. Mol Membr Biol. 2005;22:251–61.

43. NIH Human Microbiome Project - Project Catalog [Internet]. [cited 2018 Oct 9]. Available from: https://www.hmpdacc.org/catalog/

44. Chaliotis A; Vlastaridis P; Ntountoumi C; Botou M; Yalelis V; Lazou P; Tatsaki E; Mossialos D; Frillingos S; Amoutzias GD: Supporting data for "NAT/NCS2-hound: A webserver for the detection and evolutionary classification of prokaryotic and eukaryotic nucleobase-cation symporters of the NAT/NCS2 family" *GigaScience* Database. 2018. http://dx.doi.org/10.5524/100515

Figure 1

Click here to access/download;Figure;Figure1_edited.tif

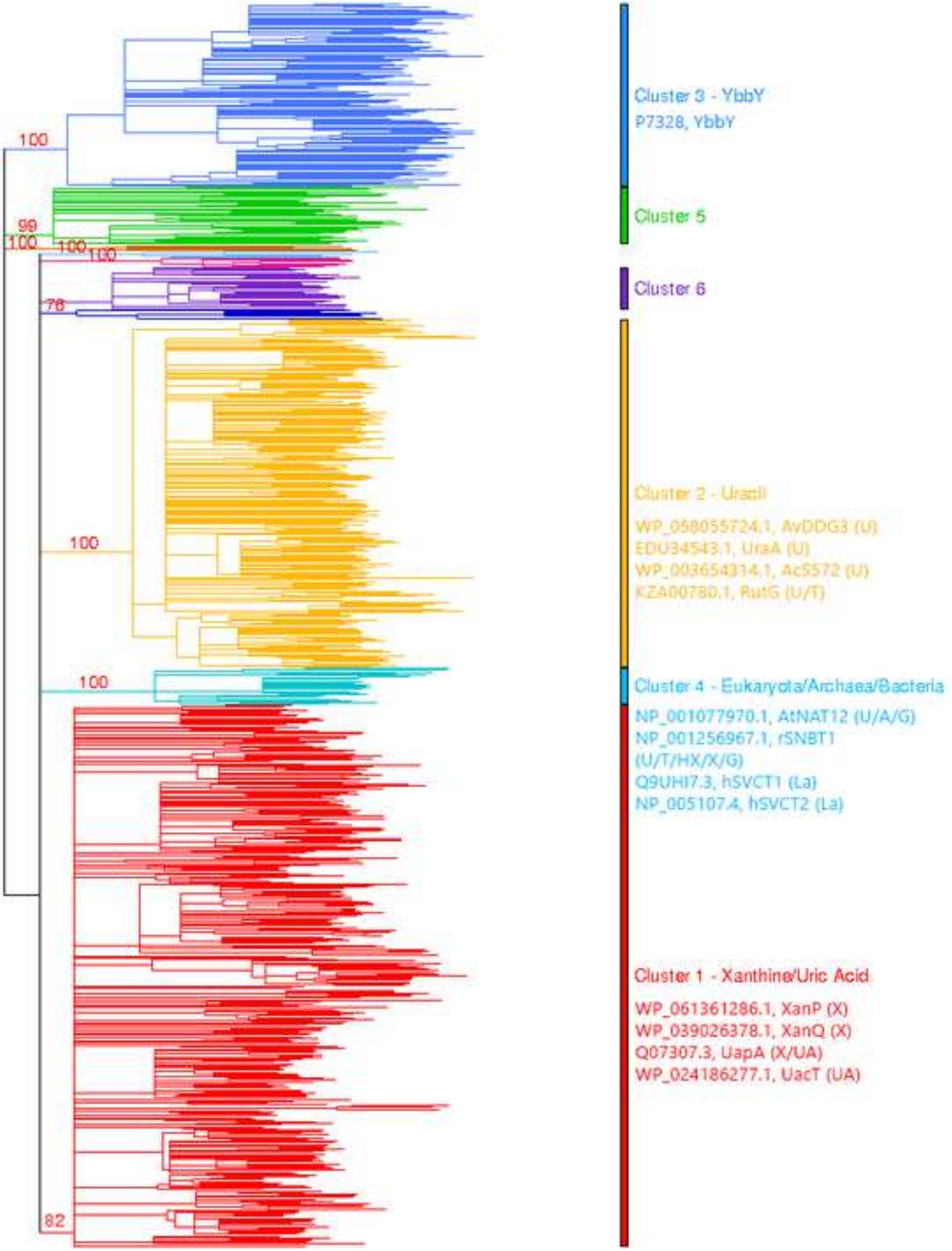

Figure 2

Click here to access/download;Figure;Fig2.png

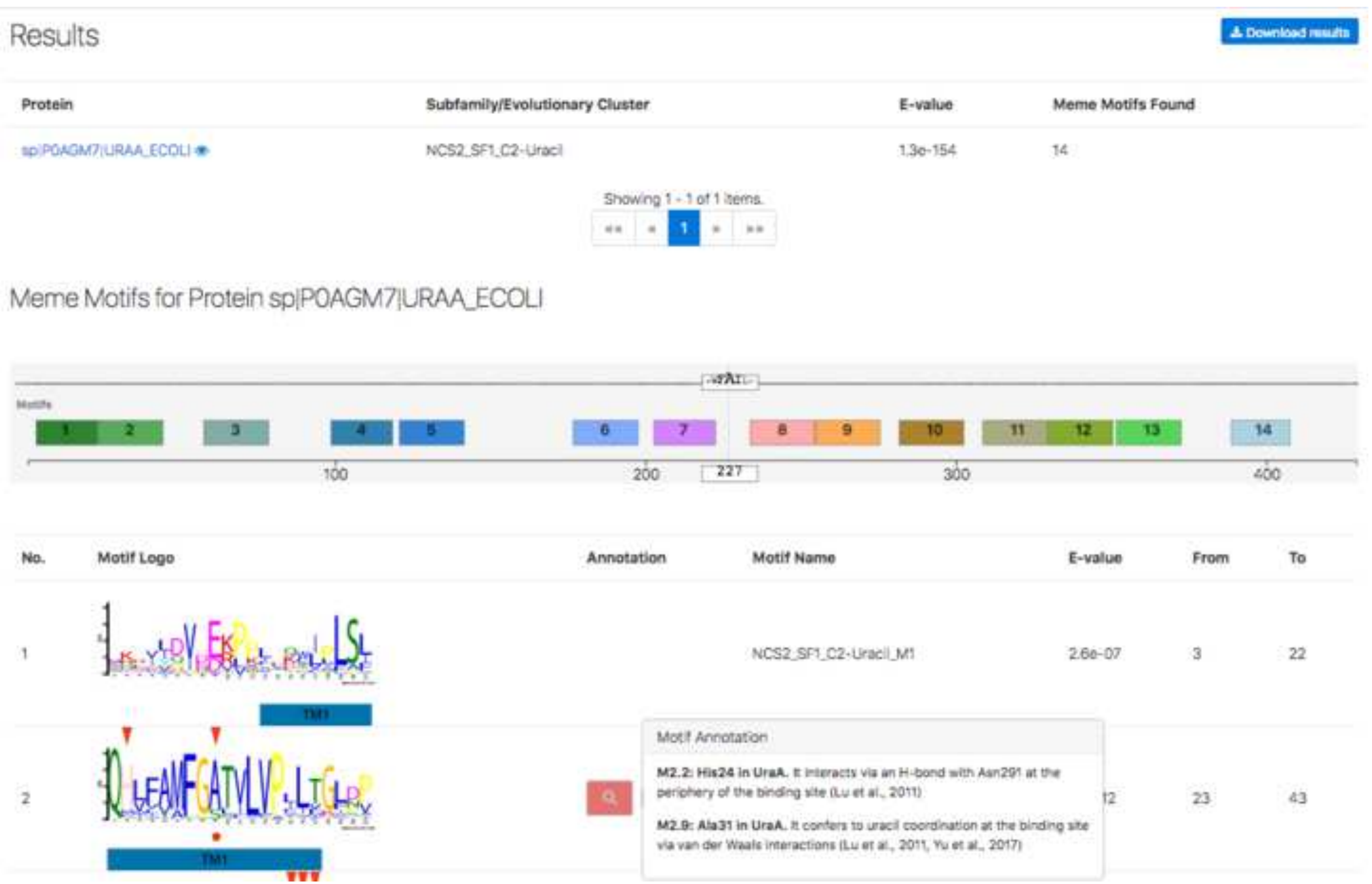

Figure 3

Click here to access/download;Figure;Fig3_proks_bo

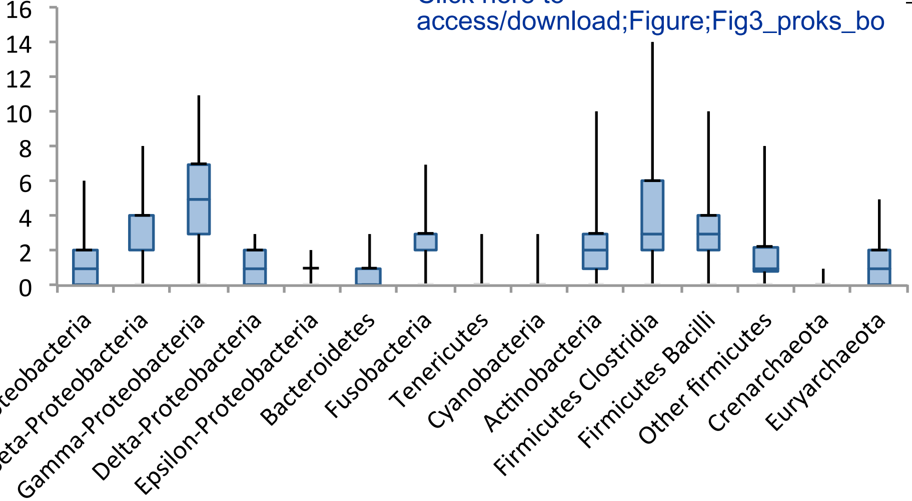

Response to Editor Click here to access/download;Personal Cover;Response_to_editor.docx

GIGA-D-18-00192R1
NAT/NCS2-hound: A webserver for the detection and evolutionary classification of prokaryotic and eukaryotic nucleobase-cation symporters of the NAT/NCS2 family.
Anargyros Chaliotis; Panayotis Vlastaridis; Chrysoula Ntountoumi; Maria Botou; Vasilis Yalelis; Panayota Lazou; Ekaterini Tatsaki; Dimitris Mossialos, Ph.D; Stathis Frillingos; Grigorios D Amoutzias, Ph.D
GigaScience

Dear Dr. Nogoy,

Thank you very much for your very positive decision and the constructive criticism we have received. We now provide a revised version of our manuscript and the supplementary data as requested by Chris Armit, for inclusion in GigaDB.

We hope that the revised version of our manuscript now addresses these essential minor formatting revisions that you have suggested/requested. We provide detailed answers to the points raised, further bellow.

We are looking forward hearing from you at your earliest convenience.

With kind regards,

Grigoris Amoutzias and Stathis Frillingos, Corresponding Authors

Grigoris Amoutzias
Assistant Professor of Bioinformatics in Genomics
Department of Biochemistry and Biotechnology
University of Thessaly
Larisa, 41500, Biopolis
Greece

Dear Dr Amoutzias,

Your manuscript "NAT/NCS2-hound: A webserver for the detection and evolutionary classification of prokaryotic and eukaryotic nucleobase-cation symporters of the NAT/NCS2 family." (GIGA-D-18-00192R1) has been assessed by our reviewers. Based on these reports, and my own assessment as Editor, I am pleased to inform you that it is potentially acceptable for publication in GigaScience, once you have carried out some essential minor formatting revisions.

1) include a diagram showing the steps in the creation of the HMMs and MEME motifs. This could also fit in protocols.io - and add the protocols.io citation to the paper.

**Answer:** We now provide a supplementary figure S12 that includes the workflow diagram that you have requested. We also mention this within the manuscript:
"A workflow of how the various HMMs and MEME motifs were generated is found in supplementary figure S12_workflow_diagram."
Concerning the protocols.io, subject, we believe/hope that the details within the Materials and Methods and the newly added workflow diagram are sufficient and protocols.io would be redundant.

2) move all URLs in the main text to the references and only cite the reference number in the main text.

**Answer:** We have now moved the URLs to the references and only cite the reference number in the main text.
"36. JHipster - Generate your Spring Boot + Angular/React applications! [Internet]. [cited 2018 Oct 9]. Available from: https://www.jhipster.tech/
37. Prediction and Evolutionary Classification Server of prokaryotic and eukaryotic NAT/NCS2 transporters [Internet]. [cited 2018 Oct 9]. Available from: http://bioinf.bio.uth.gr/nat-ncs2/
43. NIH Human Microbiome Project - Project Catalog [Internet]. [cited 2018 Oct 9]. Available from: https://www.hmpdacc.org/catalog/"

3) include a "Availability of supporting source code and requirements" section (before the data availability section)

List the following:

Project name: e.g. My bioinformatics project
Project home page: e.g. https://github.com/ISA-tools
Operating system(s): e.g. Platform independent
Programming language: e.g. Java
Other requirements: e.g. Java 1.3.1 or higher, Tomcat 4.0 or higher
License: e.g. GNU GPL, FreeBSD etc.
RRID: if applicable, e.g. RRID: SCR_014986

This needs to be under an Open Source Initiative approved license where practicable compiled running software is made available. If the code is not hosted in a repository the GigaScience GitHub repository is also available for this purpose. Their reports, together with any other comments, are below. Please also take a moment to check our website at https://giga.editorialmanager.com/ for any additional comments that were saved as attachments.

In addition, please register any new software application in the SciCrunch.org database to receive a RRID (Research Resource Identification Initiative ID) number, and include this in your manuscript. This will facilitate tracking, reproducibility and re-use of your tool.

**Answer:** We now provide this section, as requested.
Please note that we have also made some extra changes within the manuscript.
We have now acknowledged two sources of funding, that will cover our article processing costs.
In addition, Chris Armit from GigaDB has requested some modifications concerning supplementary tables that need to be provided as csv files and not excel files. We now mention these supplementary data within the manuscript accordingly.
"All results and sequence IDs are found in supplementary tables 1-6"
"...followed by many other γ-Proteobacteria (such as E.coli) with 10 members each (see supplementary tables 1 & 2)."
"10 species with 50-100 strains and 9 species had over 100 strains (see supplementary_table_3_strain_volatility.csv)."
"The number of NAT/NCS2 proteins per strain ranged from 14 to 0 (see supplementary tables 2 & 3)."
"... in bacteria of the gastrointestinal tract (see supplementary table 4)."
"... followed by Cluster 1 (14% of the total) and Cluster 2 (3% of the total) (see supplementary tables 5 and 6 for detailed results and analyzed sequences)."

The due date for submitting the revised version of your article is 15 Oct 2018.

We look forward to receiving your revised manuscript soon.

Best wishes,

Nicole Nogoy, Ph.D
GigaScience
