# Supplementary material for: NAT/NCS2-hound: a webserver for the detection and evolutionary classification of prokaryotic and eukaryotic nucleobase-cation symporters of the NAT/NCS2 family
Source: Gigascience. 2018 Nov 10;7(12):giy133. doi: 10.1093/gigascience/giy133 (PMC6308229; doi:10.1093/gigascience/giy133)
Supplement: Response_to_Reviewer_Comments_Original_Submission.pdf [file giy133_response_to_reviewer_comments_original_submission.pdf]

Author's Response To Reviewer Comments

Close

GIGA-D-18-00192
NAT/NCS2-hound: A webserver for the detection and evolutionary classification of prokaryotic and eukaryotic nucleobase-cation symporters of the NAT/NCS2 family.
Anargyros Chaliotis; Panayotis Vlastaridis; Chrysoula Ntountoumi; Maria Botou; Vasilis Yalelis; Panayota Lazou; Korina Tatsaki; Dimitris Mossialos; Stathis Frillingos; Grigorios D Amoutzias, Ph.D

GigaScience

Dear Dr. Nogoy,

We would like to thank you and the two reviewers for their constructive criticism on our submitted manuscript. We have tried to address as best as possible the concerns/corrections of the two reviewers, (see bellow). We have also registered the tool in SciCrunch.org with the RRID: SCR_016473, as requested.

We hope that you find our revised manuscript suitable for publication in Gigascience.

With kind regards,

Grigoris D. Amoutzias and Stathis Frillingos

Dear Dr Amoutzias,

Your manuscript "NAT/NCS2-hound: A webserver for the detection and evolutionary classification of prokaryotic and eukaryotic nucleobase-cation symporters of the NAT/NCS2 family." (GIGA-D-18-00192) has been assessed by our reviewers. Based on these reports, and my own assessment as Editor, I am pleased to inform you that it is potentially acceptable for publication in GigaScience, once you have carried out some essential revisions suggested by our reviewers.

We see Reviewer #1 is questioning the novelty of this web server, based on other information already available - it would be great if you can clarify their questions. The reports, together with any other comments, are below. Please also take a moment to check our website at https://giga.editorialmanager.com/ for any additional comments that were saved as attachments.

In addition, please register any new software application in the SciCrunch.org database to receive a RRID (Research Resource Identification Initiative ID) number, and include this in your manuscript. This will facilitate tracking, reproducibility and re-use of your tool.

Once you have made the necessary corrections, please submit a revised manuscript online at:

https://giga.editorialmanager.com/

If you have forgotten your username or password please use the "Send Login Details" link to get your login information. For security reasons, your password will be reset.

Please include a point-by-point within the 'Response to Reviewers' box in the submission system. Please ensure you describe additional experiments that were carried out and include a detailed rebuttal of any criticisms or requested revisions that you disagreed with. Please also ensure that your revised manuscript conforms to the journal style, which can be found in the Instructions for Authors on the journal homepage.

The due date for submitting the revised version of your article is 17 Oct 2018.

We look forward to receiving your revised manuscript soon.

Best wishes,

Nicole Nogoy, Ph.D
GigaScience
www.gigasciencejournal.com

Reviewer reports:

Reviewer #1: In this manuscript, the authors constructed a webserver for the evolutionary classification of transporters in the NAT/NCS2 family. However, I have a few concerns as below:
1. Since these transporters have long sequences and high sequence similarity, they should be easily identified from proteomes.

Although many of these transporter sequences are readily identified from proteomes, their categorization into subfamilies or association with specific substrate preferences is not easy. As a consequence, most of these transporters lack appropriate annotation in the databases (Supplementary Figures S4 and S5; and Supplementary excel file 1, spreadsheet: proteins). For subfamily 2, for example, of 592 proteins included in Figure S5, half are seen in the UNIPROT database as uncharacterized (12%) or predicted generally as permease (34%) or belonging to family NCS2 (2%) or, erroneously, SulP or MFS (2%), and half are annotated with respect to substrates, but mostly refer to general predictions for this family that are not relevant to subfamily 2 (Xanthine/Uracil/Thiamine/Ascorbate, Xanthine/Uracil/Vitamin C, Xanthine/Uracil, or Uracil) (44%) and few actually refer to substrates of the functionally known members of subfamily 2 (adenine, guanine, hypoxanthine) (6%). We now provide a user-friendly detection and categorization tool that integrated literature information and allows the specialist to better understand the nature of the NAT/NCS2 transporters of interest.

2. Moreover, these transporters have similar transmembrane structures, so the structural similarity is high. Only some of the residues on the transmembrane channel (active center) determine the specificity of the substrate and the conservation of evolution. According to these structural information, these transporters should be easily divided into subgroups.

Therefore, the innovative significance of the work is discounted.

As stated in the Introduction, "membrane transporters in general and the NAT/NCS2 family in particular are not so extensively studied to date as other categories of proteins are, due to the inherent difficulties in experimentation and in accurate prediction of their function". In the case of NAT/NCS2 family, only two members are currently known with respect to high-resolution crystal structures, UapA (subfamily 1, Cluster 1) and UraA (subfamily 1, Cluster 2). In addition, only UapA and XanQ (both in subfamily 1, Cluster 1) have been subjected to extensive mutagenesis studies to evaluate the role of all or almost all important amino acid residues, both inside and outside the substrate-binding active site. These studies (references 1, 21 and 22 of our manuscript) have indicated that major determinants of specificity are not confined in the active center but are found also at outside regions that may act as substrate-selective gates or filters. In view of the above, it is clear that the prediction of specificity is not simple and a more systematic approach (taking into account all the available sequence and experimental information) is needed. Our work provides a first step to this direction.

Overall, our work is organized in three parts: In the first one, a large-scale genome-wide evolutionary analysis identifies families and subfamilies with certain substrate preferences. In the second part, for each of these families and subfamilies we identify conserved motifs that are involved in substrate specificity. In the third part, we integrate all the above information together with experimental information to create a web tool that not only identifies NAT/NCS2 homologues in a proteome, but it also classifies them in certain families and subfamilies, it shows the conserved motifs and it also integrates the relevant functional information obtained by published experiments from other groups. This type of large-scale evolutionary analysis and user-friendly computational tool do not exist so far for the NAT/NCS2 transporters.

3. In addition, on the webpages, the substrate specificity information is not clearly reflected.

We have now added the information on the major substrate preferences of the representative functionally known homologs in each subfamily and cluster. This information is now shown every time that a query sequence is assigned to the relevant Subfamily/Evolutionary Cluster, under the heading "Known substrates", on the right side of the results page.

Reviewer #2: The Chaliotis et al provided a webserver for the detection and evolutionary classification of nucleobase-cation symporters of the NAT/NCS2 family which is of interest for the downstream users to explorer new possible symporters members.

There are a few minor comments:

1 How is the identified conserved motif within the NAT/NCS2 family shown in the homologue structure model, is it the transmembrane active site?

The identified conserved motifs correspond to transmembrane regions (TMs) from both the active site and outside the active site. In all cases, the strongest conservation was observed for a motif including residues of the predicted binding site (TM10, for motifs in subfamily 1, or TM3, for motifs in subfamily 2), followed by other motifs in the core domain (TM1, TM2 or TM9) that include residues important for the structural/functional integrity of the binding site and motifs in the gate domain (TM5 or TM12) that are implicated in the conformational

rearrangements of the alternating access mechanism (see Introduction, second paragraph). For the two subgroups for which direct structural information is available, the most well conserved motifs were in TM10 (including in UapA the xanthine-binding residues Phe406, Ala407 and Gln408) (NCS2_SF1_C1-Xanthine/Uric Acid) or in TM10 and TM3 (including in UraA the uracil-binding Tyr288, Gly289 and Glu290, and Ser72 and Phe73, respectively) (NCS2_SF1_C2-Uracil).

2 The author distinguished the Subfamily 1 into 6 clusters, I would recommend to add in the well characterized known subfamily 1 symporters or homologue in the Figure 1.

We have now revised Figure 1 and its legend, accordingly.

3 The number of the NAT/NCS2 proteins in one strain is diverse, I would expect more details about the strain specific number of NAT/NCS2 family protein and discussion regard to the physiological role of symporters in the strain.

We have now added two more paragraphs (3 and 4) in section “Distribution of the NAT/NCS2 family and subfamilies in Prokaryotes and Eukaryotes.” In paragraph 3 we discuss that for most species, the number of NAT/NCS2 proteins does not change between different strains, but we also highlight the most notable exceptions. We have also added in supplementary file 1 another worksheet (strain volatility), where we show how the number of NAT/NCS2 transporters changes from minimum to maximum among strains of that species. Thus, the reader may further explore this particular subject. In paragraph 4 we discuss the biological significance of the number of NAT/NCS2 proteins in the various strains/organisms.

Close
