# Supplementary material for: NAT/NCS2-hound: a webserver for the detection and evolutionary classification of prokaryotic and eukaryotic nucleobase-cation symporters of the NAT/NCS2 family
Source: Gigascience. 2018 Nov 10;7(12):giy133. doi: 10.1093/gigascience/giy133 (PMC6308229; doi:10.1093/gigascience/giy133)
Supplement: Response_to_Reviewer_Comments_revision_1.pdf [file giy133_response_to_reviewer_comments_revision_1.pdf]

Author's Response To Reviewer Comments

Close

GIGA-D-18-00192R1
NAT/NCS2-hound: A webserver for the detection and evolutionary classification of prokaryotic and eukaryotic nucleobase-cation symporters of the NAT/NCS2 family.
Anargyros Chaliotis; Panayotis Vlastaridis; Chrysoula Ntountoumi; Maria Botou; Vasilis Yalelis; Panayota Lazou; Ekaterini Tatsaki; Dimitris Mossialos, Ph.D; Stathis Frillingos; Grigorios D Amoutzias, Ph.D
GigaScience

Dear Dr. Nogoy,

Thank you very much for your very positive decision and the constructive criticism we have received. We now provide a revised version of our manuscript and the supplementary data as requested by Chris Armit, for inclusion in GigaDB.

We hope that the revised version of our manuscript now addresses these essential minor formatting revisions that you have suggested/requested. We provide detailed answers to the points raised, further bellow.

We are looking forward hearing from you at your earliest convenience.

With kind regards,

Grigoris Amoutzias and Stathis Frillingos, Corresponding Authors

Grigoris Amoutzias
Assistant Professor of Bioinformatics in Genomics
Department of Biochemistry and Biotechnology
University of Thessaly
Larisa, 41500, Biopolis
Greece

Dear Dr Amoutzias,

Your manuscript "NAT/NCS2-hound: A webserver for the detection and evolutionary classification of prokaryotic and eukaryotic nucleobase-cation symporters of the NAT/NCS2 family." (GIGA-D-18-00192R1) has been assessed by our reviewers. Based on these reports, and my own assessment as Editor, I am pleased to inform you that it is potentially acceptable for publication in GigaScience, once you have carried out some essential minor formatting revisions.

1) include a diagram showing the steps in the creation of the HMMs and MEME motifs. This could also fit in protocols.io - and add the protocols.io citation to the paper.

Answer: We now provide a supplementary figure S12 that includes the workflow diagram

that you have requested. We also mention this within the manuscript:
“A workflow of how the various HMMs and MEME motifs were generated is found in supplementary figure S12_workflow_diagram.”
Concerning the protocols.io, subject, we believe/hope that the details within the Materials and Methods and the newly added workflow diagram are sufficient and protocols.io would be redundant.

2) move all URLs in the main text to the references and only cite the reference number in the main text.

Answer: We have now moved the URLs to the references and only cite the reference number in the main text.
“36. JHipster - Generate your Spring Boot + Angular/React applications! [Internet]. [cited 2018 Oct 9]. Available from: https://www.jhipster.tech/
37. Prediction and Evolutionary Classification Server of prokaryotic and eukaryotic NAT/NCS2 transporters [Internet]. [cited 2018 Oct 9]. Available from: http://bioinf.bio.uth.gr/nat-ncs2/
43. NIH Human Microbiome Project - Project Catalog [Internet]. [cited 2018 Oct 9]. Available from: https://www.hmpdacc.org/catalog/”

3) include a "Availability of supporting source code and requirements" section (before the data availability section)

List the following:

Project name: e.g. My bioinformatics project
Project home page: e.g. https://github.com/ISA-tools
Operating system(s): e.g. Platform independent
Programming language: e.g. Java
Other requirements: e.g. Java 1.3.1 or higher, Tomcat 4.0 or higher
License: e.g. GNU GPL, FreeBSD etc.
RRID: if applicable, e.g. RRID: SCR_014986

This needs to be under an Open Source Initiative approved license where practicable compiled running software is made available. If the code is not hosted in a repository the GigaScience GitHub repository is also available for this purpose.
Their reports, together with any other comments, are below. Please also take a moment to check our website at https://giga.editorialmanager.com/ for any additional comments that were saved as attachments.

In addition, please register any new software application in the SciCrunch.org database to receive a RRID (Research Resource Identification Initiative ID) number, and include this in your manuscript. This will facilitate tracking, reproducibility and re-use of your tool.

Answer: We now provide this section, as requested.
Please note that we have also made some extra changes within the manuscript.
We have now acknowledged two sources of funding, that will cover our article processing costs.
In addition, Chris Armit from GigaDB has requested some modifications concerning supplementary tables that need to be provided as csv files and not excel files. We now

mention these supplementary data within the manuscript accordingly.
“All results and sequence IDs are found in supplementary tables 1-6”
“…followed by many other γ-Proteobacteria (such as E.coli) with 10 members each (see supplementary tables 1 & 2).”
“10 species with 50-100 strains and 9 species had over 100 strains (see supplementary_table_3_strain_volatility.csv).”
“The number of NAT/NCS2 proteins per strain ranged from 14 to 0 (see supplementary tables 2 & 3).”
“… in bacteria of the gastrointestinal tract (see supplementary table 4).”
“… followed by Cluster 1 (14% of the total) and Cluster 2 (3% of the total) (see supplementary tables 5 and 6 for detailed results and analyzed sequences).”

The due date for submitting the revised version of your article is 15 Oct 2018.

We look forward to receiving your revised manuscript soon.

Best wishes,

Nicole Nogoy, Ph.D
GigaScience

Close
